# Supplementary material for: Strong correlational but no causal evidence on the link between the perception of scientific consensus and support for vaccination
Source: PLoS One. 2024 Jan 3;19(1):e0296066. doi: 10.1371/journal.pone.0296066 (PMC10763927; doi:10.1371/journal.pone.0296066)
Supplement: S1 File — (DOCX) [file pone.0296066.s001.docx]

Supporting information for “Strong correlational but no causal evidence on the link between the perception of scientific consensus and support for vaccination”

Contents

[S1. Study 1 3](#_Toc133594696)

[S1.1. Effects of perceived consensus on vaccine attitudes 3](#_Toc133594697)

[S1.2. Testing hypotheses with SEM 4](#_Toc133594698)

[S1.3. Effects of perceived consensus and moderators on vaccine attitudes 6](#_Toc133594699)

[S2. Study 2 10](#_Toc133594700)

[S2.1. Testing hypotheses with OLS regression 10](#_Toc133594701)

[S2.1.1. The effects of consensus messaging on perception of scientific consensus 10](#_Toc133594702)

[S2.1.2. The effects of consensus messaging on vaccine attitudes 12](#_Toc133594703)

[S2.2. Testing hypotheses with SEM 16](#_Toc133594704)

[S2.2.1. Wave 1 17](#_Toc133594705)

[S2.2.2. Wave 2 19](#_Toc133594706)

[S3. Study 3 23](#_Toc133594707)

[S3.1. Analysis of sensitization effects 23](#_Toc133594708)

[S3.2. Testing hypotheses with OLS regression 25](#_Toc133594709)

[S3.2.1. The effects of consensus messaging on perception of scientific consensus 25](#_Toc133594710)

[S3.2.2. The effects of consensus messaging on vaccine attitudes and intentions 28](#_Toc133594711)

[S3.2.3. Moderators of the effects of consensus messaging on vaccine attitudes and intentions 30](#_Toc133594712)

[S3.2.4 Moderators of the effects of consensus messaging on COVID-19 vaccination intentions 34](#_Toc133594713)

[S3.3. Testing hypotheses with SEM 39](#_Toc133594714)

[S3.3.1. Pre-post design condition 40](#_Toc133594715)

[S3.3.2. Post-only design condition 44](#_Toc133594716)

[S3.4. Analysis of psychological reactance 48](#_Toc133594717)

[S3.4.1. The effects of consensus messaging and moderator on psychological reactance 48](#_Toc133594718)

[S3.4.2. The effects of psychological reactance and consensus messaging on the main DVs 52](#_Toc133594719)

# **S1. Study 1**

## **S1.1. Effects of perceived consensus on vaccine attitudes**

S1a Table. *The association between the* ***perceived scientific consensus*** *and vaccine worry, belief, and policy support in Study 1*

|  | **Worry** | | | | **Belief** | | | | **Policy support** | | | |
| --- | --- | --- | --- | --- | --- | --- | --- | --- | --- | --- | --- | --- |
|  | *b* | *SE* | *t* | *p* | *b* | *SE* | *t* | *p* | *b* | *SE* | *t* | *p* |
| Intercept | 0.54 | 0.02 | 23.52 | **<0.001** | 0.53 | 0.02 | 23.20 | **<0.001** | 0.41 | 0.03 | 15.03 | **<0.001** |
| Age | -0.13 | 0.03 | -4.06 | **<0.001** | 0.20 | 0.03 | 6.39 | **<0.001** | 0.37 | 0.04 | 9.94 | **<0.001** |
| Education | -0.00 | 0.03 | -0.15 | 0.883 | -0.00 | 0.03 | -0.16 | 0.873 | -0.06 | 0.03 | -1.78 | 0.075 |
| Gender [Women] | -0.04 | 0.02 | -2.23 | **0.026** | 0.01 | 0.02 | 0.75 | 0.451 | -0.00 | 0.02 | -0.20 | 0.842 |
| Perceived scientific consensus | -0.79 | 0.03 | -24.33 | **<0.001** | 0.76 | 0.03 | 23.73 | **<0.001** | 0.84 | 0.04 | 21.91 | **<0.001** |
| R^2^ / R^2^ adjusted | 0.501 / 0.498 | | | | 0.501 / 0.498 | | | | - 1. / 0.492 | | | |

S1b Table. *The association between the* ***perceived scientific consensus*** *and vaccine worry, belief, and policy support in Study 1 (subjects with low consensus perception are included; we do not control for demographic variables).*

|  | **Worry** | | | | **Beliefs** | | | | **Policy support** | | | |
| --- | --- | --- | --- | --- | --- | --- | --- | --- | --- | --- | --- | --- |
|  | *b* | *SE* | *t* | *p* | *b* | *SE* | *t* | *p* | *b* | *SE* | *t* | *p* |
| Intercept | 0.46 | 0.01 | 56.37 | **<0.001** | 0.62 | 0.01 | 76.37 | **<0.001** | 0.55 | 0.01 | 54.31 | **<0.001** |
| Perceived scientific consensus | -0.80 | 0.03 | -25.50 | **<0.001** | 0.81 | 0.03 | 25.85 | **<0.001** | 0.90 | 0.04 | 23.12 | **<0.001** |
| R^2^ / R^2^ adjusted | 0.480 / 0.480 | | | | 0.487 / 0.487 | | | | 0.432 / 0.431 | | | |

## **S1.2. Testing hypotheses with SEM**

To test for the full Gateway Belief Model, we fit structural equation model (SEM) testing paths between perceived scientific consensus, through belief and worry, on policy support. This model is based on previous work on scientific consensus (Kerr & van der Linden, 2021). This model was fit using the ML estimator and parameters were estimated with model-based bootstrapping (with 5,000 iterations). All variables were also rescaled between 0 and 1 prior to analyses. The model had a good fit (χ^2^(1) = 0.1, p = .671, CFI = 1.000, SRMR = 0.002, RMSEA = 0.000, 95%CI[0.000, 0.083]). As shown in S2, all of the reported paths are statistically significant. The effects of perceived scientific consensus are in line with the above-presented OLS regression (e.g., positive associations between perceived consensus and COVID-19 attitudes). What is new here are the associations between the DVs (i.e., worry, belief, policy support) and the indirect effects. This analysis showed that while belief is negatively associated with worry, and positively with policy support, worry is negatively related to policy support. All indirect effects of perceived scientific consensus on policy support (i.e., through belief, worry, and both belief and worry) were positive.

S2 Table. *Path coefficients and indirect effects in Study 1.*

|  |  |  |  |  |  |  |  | 95% CI | |
| --- | --- | --- | --- | --- | --- | --- | --- | --- | --- |
| Paths |  |  |  | *b* | SE | *z* | *p* | Lower | Upper |
| Perceived scientific consensus | → | Belief |  | 0.80 | 0.03 | 26.70 | <.001 | 0.74 | 0.86 |
| Perceived scientific consensus | → | Worry |  | -0.37 | 0.04 | -8.40 | <.001 | -0.45 | -0.29 |
| Belief | → | Worry |  | -0.56 | 0.04 | -14.70 | <.001 | -0.63 | -0.48 |
| Belief | → | Policy support |  | 0.71 | 0.04 | 16.99 | <.001 | 0.62 | 0.79 |
| Worry | → | Policy support |  | -0.40 | 0.04 | -9.20 | <.001 | -0.48 | -0.31 |
| *Indirect effect* |  |  |  |  |  |  | <.001 |  |  |
| Through belief |  |  |  | 0.57 | 0.04 | 14.08 | <.001 | 0.49 | 0.65 |
| Through worry |  |  |  | 0.15 | 0.02 | 6.11 | <.001 | 0.11 | 0.20 |
| Through belief and worry |  |  |  | 0.18 | 0.02 | 7.57 | <.001 | 0.14 | 0.23 |
| Combined indirect effect |  |  |  | 0.89 | 0.03 | 30.22 | <.001 | 0.83 | 0.95 |

*Note.* Indirect effects refer to effects of perceived scientific consensus on policy support.

## **S1.3. Effects of perceived consensus and moderators on vaccine attitudes**

S3a Table. ***The moderating effects of ideology*** *on the association between perceived scientific and vaccine worry, belief, and policy support in Study 1*

|  | **Worry** | | | | **Belief** | | | | **Policy support** | | | |
| --- | --- | --- | --- | --- | --- | --- | --- | --- | --- | --- | --- | --- |
|  | *b* | *SE* | *t* | *p* | *b* | *SE* | *t* | *p* | *b* | *SE* | *t* | *p* |
| Intercept | 0.53 | 0.02 | 23.31 | **<0.001** | 0.53 | 0.02 | 23.37 | **<0.001** | 0.42 | 0.03 | 15.40 | **<0.001** |
| Age | -0.12 | 0.03 | -3.86 | **<0.001** | 0.20 | 0.03 | 6.29 | **<0.001** | 0.37 | 0.04 | 9.82 | **<0.001** |
| Education | 0.02 | 0.03 | 0.70 | 0.482 | -0.01 | 0.03 | -0.53 | 0.594 | -0.08 | 0.03 | -2.40 | **0.017** |
| Gender [Women] | -0.04 | 0.02 | -2.41 | **0.016** | 0.01 | 0.02 | 0.86 | 0.390 | -0.00 | 0.02 | -0.09 | 0.930 |
| Perceived scientific consensus | -0.78 | 0.03 | -23.33 | **<0.001** | 0.75 | 0.03 | 22.37 | **<0.001** | 0.83 | 0.04 | 20.85 | **<0.001** |
| Ideology | 0.13 | 0.03 | 3.76 | **<0.001** | -0.08 | 0.03 | -2.28 | **0.023** | -0.12 | 0.04 | -2.90 | **0.004** |
| Perceived scientific consensus x Ideology | 0.33 | 0.13 | 2.65 | **0.008** | -0.04 | 0.13 | -0.30 | 0.766 | -0.27 | 0.15 | -1.81 | 0.071 |
| R^2^ / R^2^ adjusted | 0.517 / 0.513 | | | | 0.505 / 0.501 | | | | 0.504 / 0.499 | | | |


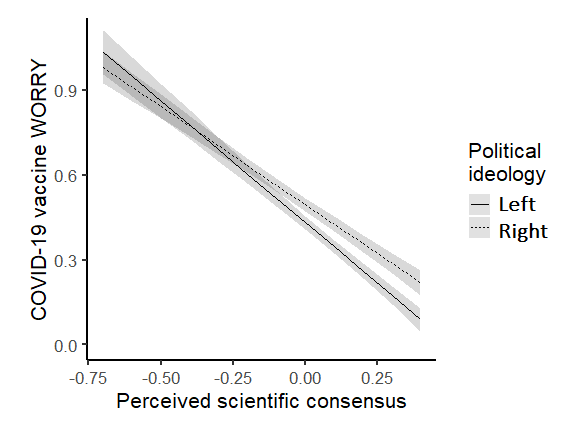


S1 Fig. The effects of perceived consensus and political ideology on vaccine worry.

S3b Table. ***The moderating effects of ideology*** *on the association between perceived scientific and vaccine worry, belief, and policy support in Study 1 (subjects with low consensus perception are included; we do not control for demographic variables).*

|  | **Worry** | | | | **Beliefs** | | | | **Policy support** | | | |
| --- | --- | --- | --- | --- | --- | --- | --- | --- | --- | --- | --- | --- |
|  | *b* | *SE* | *t* | *p* | *b* | *SE* | *t* | *p* | *b* | *SE* | *t* | *p* |
| Intercept | 0.47 | 0.01 | 56.29 | **<0.001** | 0.62 | 0.01 | 74.51 | **<0.001** | 0.54 | 0.01 | 52.93 | **<0.001** |
| Perceived scientific consensus | -0.78 | 0.03 | -24.26 | **<0.001** | 0.79 | 0.03 | 24.39 | **<0.001** | 0.88 | 0.04 | 21.91 | **<0.001** |
| Ideology | 0.13 | 0.03 | 3.92 | **<0.001** | -0.08 | 0.03 | -2.41 | **0.016** | -0.12 | 0.04 | -2.94 | **0.003** |
| Perceived scientific consensus x Ideology | 0.30 | 0.13 | 2.41 | **0.016** | -0.06 | 0.13 | -0.49 | 0.622 | -0.27 | 0.16 | -1.71 | 0.087 |
| R^2^ / R^2^ adjusted | 0.498 / 0.495 | | | | 0.492 / 0.490 | | | | 0.442 / 0.440 | | | |

S4a Table. ***The moderating effects of trust in scientists*** *on the association between perceived scientific and vaccine worry, belief, and policy support in Study 1*

|  | **Worry** | | | | **Beliefs** | | | | **Policy support** | | | |
| --- | --- | --- | --- | --- | --- | --- | --- | --- | --- | --- | --- | --- |
|  | *b* | *SE* | *t* | *p* | *b* | *SE* | *t* | *p* | *b* | *SE* | *t* | *p* |
| Intercept | 0.50 | 0.02 | 23.70 | **<0.001** | 0.58 | 0.02 | 27.98 | **<0.001** | 0.47 | 0.02 | 19.19 | **<0.001** |
| Age | -0.06 | 0.03 | -1.93 | 0.055 | 0.13 | 0.03 | 4.61 | **<0.001** | 0.28 | 0.03 | 8.39 | **<0.001** |
| Education | 0.04 | 0.02 | 1.49 | 0.136 | -0.04 | 0.02 | -1.90 | 0.058 | -0.11 | 0.03 | -3.90 | **<0.001** |
| Gender [Women] | -0.02 | 0.01 | -1.63 | 0.104 | 0.00 | 0.01 | 0.05 | 0.959 | -0.02 | 0.02 | -1.14 | 0.254 |
| Perceived scientific consensus | -0.56 | 0.04 | -13.10 | **<0.001** | 0.46 | 0.04 | 11.07 | **<0.001** | 0.49 | 0.05 | 10.12 | **<0.001** |
| Trust in scientists | -0.44 | 0.04 | -11.89 | **<0.001** | 0.50 | 0.04 | 13.62 | **<0.001** | 0.61 | 0.04 | 14.18 | **<0.001** |
| Perceived scientific consensus x Trust | -0.49 | 0.11 | -4.65 | **<0.001** | 0.26 | 0.10 | 2.56 | **0.011** | 0.48 | 0.12 | 4.02 | **<0.001** |
| Observations | 698 | | | | 698 | | | | 698 | | | |
| R^2^ / R^2^ adjusted | 0.596 / 0.593 | | | | 0.609 / 0.606 | | | | 0.615 / 0.612 | | | |

*Simple slopes analysis for trust in scientists and consensus perception interaction*

For worry, the effects of perceived scientific consensus among those who trust in scientists (b = -0.68, SE = 0.06, 95%CI[-0.79, -0.56]) were stronger than for those low on trust (b = -0.43, SE = 0.04, 95%CI[-0.51, -0.35]), and this difference was statistically significant (b = 0.26, SE = 0.06, t(691) = 4.65, p <.001); the model predictions are shown in Fig 1 (Panel A) in the main text. For belief, again, the effects of perceived consensus were stronger among those who trust in scientists (b = 0.53, SE = 0.06, 95%CI[0.41, 0.63]) as compared to those who do not (b = 0.39, SE = 0.04, 95%CI[0.31, 0.47]), and this difference was significant (b = 0.14, SE = 0.06, t(691) = 2.56, p = .012); the model predictions are shown in Fig 1 (Panel B). Similarly, for policy support, the effects of perceived consensus were stronger for those who trust scientists (b = 0.62, SE = 0.07, 95%CI[0.49, 0.76]) compared to those who do not (b = 0.36, SE = 0.05, 95%CI[0.27, 0.45]) and the difference was significant (b = 0.26, SE = 0.07, t(691) = 4.02, p <.001); the model predictions are shown in Fig 1 (Panel C).

S4b Table. ***The moderating effects of trust in scientists*** *on the association between perceived scientific and vaccine worry, belief, and policy support in Study 1 (subjects with low consensus perception are included; we do not control for demographic variables).*

|  | **Worry** | | | | **Beliefs** | | | | **Policy support** | | | |
| --- | --- | --- | --- | --- | --- | --- | --- | --- | --- | --- | --- | --- |
|  | *b* | *SE* | *t* | *p* | *b* | *SE* | *t* | *p* | *b* | *SE* | *t* | *p* |
| Intercept | 0.49 | 0.01 | 55.32 | **<0.001** | 0.61 | 0.01 | 69.85 | **<0.001** | 0.52 | 0.01 | 48.81 | **<0.001** |
| Perceived scientific consensus | -0.56 | 0.04 | -13.23 | **<0.001** | 0.48 | 0.04 | 11.46 | **<0.001** | 0.52 | 0.05 | 10.17 | **<0.001** |
| Trust in scientists | -0.45 | 0.04 | -12.03 | **<0.001** | 0.51 | 0.04 | 14.16 | **<0.001** | 0.64 | 0.04 | 14.46 | **<0.001** |
| Perceived scientific consensus x Trust | -0.59 | 0.10 | -5.83 | **<0.001** | 0.31 | 0.10 | 3.19 | **0.001** | 0.61 | 0.12 | 5.07 | **<0.001** |
| R^2^ / R^2^ adjusted | 0.589 / 0.587 | | | | 0.608 / 0.606 | | | | 0.577 / 0.576 | | | |

# S2. Study 2

## **S2.1. Testing hypotheses with OLS regression**

### **S2.1.1. The effects of consensus messaging on perception of scientific consensus**

S5a Table. *The effects of consensus messaging and moderators on perception of consensus in wave 1 and wave 2 in Study 2*

|  | **Wave 1** | | | **Wave 2** | | | **Wave 1: Ideology** | | | **Wave 2: Ideology** | | | **Wave 1: Trust** | | | **Wave 2: Trust** | | |
| --- | --- | --- | --- | --- | --- | --- | --- | --- | --- | --- | --- | --- | --- | --- | --- | --- | --- | --- |
|  | *b* | *SE* | *p* | *b* | *SE* | *p* | *b* | *SE* | *p* | *b* | *SE* | *p* | *b* | *SE* | *p* | *b* | *SE* | *p* |
| Intercept | 8.56 | 1.87 | **<0.001** | 8.49 | 2.25 | **<0.001** | 8.06 | 1.90 | **<0.001** | 8.57 | 2.32 | **<0.001** | 5.62 | 1.92 | **0.004** | 5.97 | 2.32 | **0.010** |
| Age | -8.57 | 2.38 | **<0.001** | -11.71 | 2.78 | **<0.001** | -8.14 | 2.40 | **0.001** | -11.68 | 2.83 | **<0.001** | -5.98 | 2.37 | **0.012** | -9.10 | 2.79 | **0.001** |
| Education | -3.53 | 1.97 | 0.074 | -4.77 | 2.28 | **0.037** | -3.05 | 2.01 | 0.128 | -4.87 | 2.34 | **0.038** | -1.49 | 1.97 | 0.450 | -3.41 | 2.29 | 0.138 |
| Gender | -0.30 | 1.20 | 0.799 | 2.37 | 1.37 | 0.083 | -0.32 | 1.20 | 0.787 | 2.41 | 1.37 | 0.079 | 0.78 | 1.18 | 0.511 | 3.35 | 1.36 | **0.014** |
| Condition [Consensus] | 9.64 | 1.46 | **<0.001** | 6.80 | 1.67 | **<0.001** | 9.70 | 1.46 | **<0.001** | 6.74 | 1.68 | **<0.001** | 9.93 | 1.42 | **<0.001** | 7.10 | 1.65 | **<0.001** |
| Condition [Consensus & pluralism] | 9.00 | 1.44 | **<0.001** | 5.57 | 1.66 | **0.001** | 9.04 | 1.44 | **<0.001** | 5.53 | 1.67 | **0.001** | 9.23 | 1.41 | **<0.001** | 6.04 | 1.64 | **<0.001** |
| Ideology |  |  |  |  |  |  | 1.50 | 4.22 | 0.723 | -3.23 | 5.05 | 0.523 |  |  |  |  |  |  |
| Ideology x Condition [Consensus] |  |  |  |  |  |  | -1.02 | 5.87 | 0.863 | 3.14 | 6.90 | 0.649 |  |  |  |  |  |  |
| Ideology x Condition [Consensus & pluralism] |  |  |  |  |  |  | 6.19 | 5.69 | 0.276 | 4.33 | 6.67 | 0.517 |  |  |  |  |  |  |
| Trust in scientists |  |  |  |  |  |  |  |  |  |  |  |  | -2.80 | 3.87 | 0.470 | -0.71 | 4.48 | 0.874 |
| Trust in scientists x Condition [Consensus] |  |  |  |  |  |  |  |  |  |  |  |  | -15.89 | 5.38 | **0.003** | -15.30 | 6.27 | **0.015** |
| Trust in scientists x Condition [Consensus & pluralism] |  |  |  |  |  |  |  |  |  |  |  |  | -11.94 | 5.35 | **0.026** | -14.71 | 6.31 | **0.020** |
| Observations | 611 | | | 477 | | | 611 | | | 477 | | | 611 | | | 477 | | |
| R^2^ / R^2^ adjusted | 0.109 / 0.101 | | | 0.084 / 0.074 | | | 0.115 / 0.103 | | | 0.084 / 0.069 | | | 0.158 / 0.147 | | | 0.126 / 0.111 | | |

S5b Table. *The effects of consensus messaging and moderators on perception of consensus in wave 1 and wave 2 in Study 2 (subjects with low consensus perception, low credibility of manipulation perception, and speeders are included; we do not control for demographic variables).*

|  | **Wave 1** | | | **Wave 2** | | | **Wave 1: Ideology** | | | **Wave 2: Ideology** | | | **Wave 1: Trust** | | | **Wave 2: Trust** | | |
| --- | --- | --- | --- | --- | --- | --- | --- | --- | --- | --- | --- | --- | --- | --- | --- | --- | --- | --- |
|  | *b* | *SE* | *p* | *b* | *SE* | *p* | *b* | *SE* | *p* | *b* | *SE* | *p* | *b* | *SE* | *p* | *b* | *SE* | *p* |
| Intercept | 2.90 | 1.25 | **0.021** | 1.29 | 1.36 | 0.343 | 2.88 | 1.24 | **0.021** | 1.30 | 1.36 | 0.337 | 2.90 | 1.20 | **0.016** | 1.30 | 1.32 | 0.326 |
| Condition [Consensus] | 12.41 | 1.72 | **<0.001** | 8.61 | 1.86 | **<0.001** | 12.46 | 1.71 | **<0.001** | 8.61 | 1.86 | **<0.001** | 12.12 | 1.65 | **<0.001** | 8.44 | 1.81 | **<0.001** |
| Condition [Consensus&pluralism] | 11.23 | 1.74 | **<0.001** | 7.51 | 1.89 | **<0.001** | 11.32 | 1.74 | **<0.001** | 7.60 | 1.89 | **<0.001** | 11.52 | 1.67 | **<0.001** | 8.12 | 1.84 | **<0.001** |
| Ideology |  |  |  |  |  |  | 2.45 | 5.08 | 0.630 | -3.35 | 5.71 | 0.557 |  |  |  |  |  |  |
| Ideology x Condition [Consensus] |  |  |  |  |  |  | 9.53 | 6.94 | 0.170 | 12.11 | 7.64 | 0.113 |  |  |  |  |  |  |
| Ideology x Condition [Consensus&pluralism] |  |  |  |  |  |  | 7.06 | 6.78 | 0.298 | 9.98 | 7.51 | 0.185 |  |  |  |  |  |  |
| Trust in scientists |  |  |  |  |  |  |  |  |  |  |  |  | -3.97 | 4.17 | 0.341 | -0.95 | 4.65 | 0.838 |
| Trust in scientists x Condition [Consensus] |  |  |  |  |  |  |  |  |  |  |  |  | -19.36 | 5.65 | **0.001** | -16.51 | 6.20 | **0.008** |
| Trust in scientists x Condition [Consensus&pluralism] |  |  |  |  |  |  |  |  |  |  |  |  | -19.02 | 5.76 | **0.001** | -19.44 | 6.36 | **0.002** |
| Observations | 753 | | | 586 | | | 753 | | | 586 | | | 753 | | | 586 | | |
| R^2^ / R^2^ adjusted | 0.076 / 0.074 | | | 0.041 / 0.037 | | | 0.090 / 0.084 | | | 0.049 / 0.041 | | | 0.157 / 0.151 | | | 0.103 / 0.095 | | |

### **S2.1.2. The effects of consensus messaging on vaccine attitudes**

S6a Table. *The effects of consensus messaging on vaccine belief, worry, and policy support in wave 1 and wave 2 in Study 2*

|  | **Wave 1: Belief** | | | **Wave 2: Belief** | | | **Wave 1: Worry** | | | **Wave 2: Worry** | | | **Wave 1: Policy support** | | | **Wave 2: Policy support** | | |
| --- | --- | --- | --- | --- | --- | --- | --- | --- | --- | --- | --- | --- | --- | --- | --- | --- | --- | --- |
|  | *b* | *SE* | *p* | *b* | *SE* | *p* | *b* | *SE* | *p* | *b* | *SE* | *p* | *b* | *SE* | *p* | *b* | *SE* | *p* |
| Intercept | 0.11 | 0.06 | 0.087 | 0.07 | 0.10 | 0.456 | 0.03 | 0.07 | 0.670 | -0.15 | 0.10 | 0.152 | -0.05 | 0.05 | 0.272 | 0.12 | 0.08 | 0.132 |
| Age | -0.08 | 0.08 | 0.340 | -0.01 | 0.12 | 0.918 | -0.01 | 0.09 | 0.930 | -0.09 | 0.13 | 0.455 | 0.09 | 0.06 | 0.115 | -0.06 | 0.10 | 0.535 |
| Education | 0.02 | 0.07 | 0.747 | -0.06 | 0.10 | 0.515 | -0.12 | 0.08 | 0.111 | 0.05 | 0.10 | 0.657 | -0.03 | 0.05 | 0.589 | -0.05 | 0.08 | 0.508 |
| Gender | 0.01 | 0.04 | 0.783 | -0.01 | 0.06 | 0.904 | -0.02 | 0.05 | 0.655 | 0.00 | 0.06 | 0.942 | -0.01 | 0.03 | 0.692 | 0.03 | 0.05 | 0.587 |
| Condition [Consensus] | -0.10 | 0.05 | **0.039** | 0.01 | 0.07 | 0.896 | 0.05 | 0.06 | 0.408 | 0.05 | 0.08 | 0.551 | -0.01 | 0.04 | 0.848 | -0.05 | 0.06 | 0.443 |
| Condition [Consensus & pluralism] | 0.03 | 0.05 | 0.525 | 0.12 | 0.07 | 0.086 | 0.02 | 0.05 | 0.703 | 0.21 | 0.08 | **0.007** | 0.06 | 0.04 | 0.100 | -0.03 | 0.06 | 0.601 |
| Observations | 611 | | | 477 | | | 611 | | | 477 | | | 611 | | | 477 | | |
| R^2^ / R^2^ adjusted | 0.015 / 0.007 | | | 0.009 / -0.002 | | | 0.006 / -0.002 | | | 0.018 / 0.008 | | | 0.011 / 0.003 | | | 0.003 / -0.007 | | |

*Analysis of the effects of manipulation of attitudes*

As mentioned in the main text, we found a main effect of consensus messaging on vaccine belief at wave 1 (F(2,605) = 3.97, p = .019) and on worry at wave 2 (F(2,471) = 4.08, p = .017); however, the patterns of the results were unexpected in both cases. With regard to belief at wave 1, we found that belief became more positive in the consensus & pluralism, and in the control conditions, whereas they were near-zero in the consensus condition, as presented in S2 Fig (Panel A). Pair-wise comparisons showed that a change in belief was smaller in the consensus condition as compared to the consensus & pluralism condition (b = -0.14, SE = 0.05, t(605) = -2.70, p = .019), but the comparison against the control condition was non-significant (b = 0.10, SE = 0.05, t(605) = 2.07, p = .097); the comparison between consensus & pluralism, and the control condition was also non-significant (b = -0.03, SE = 0.05, t(605) = -0.64, p = .801).

Secondly, we found that vaccine worry decreased at wave 2 (as the estimates are below 0) in the control, and in the consensus conditions, whereas it was near-zero in the consensus & pluralism condition as shown in S2 Fig (Panel B). Pair-wise comparisons showed that differences between consensus & pluralism vs. the control condition were statistically significant (b = -0.21, SE = 0.08, t(471) = -2.72, p = .019) and non-significant for the comparison between consensus & pluralism and consensus condition (b = -0.16, SE = 0.08, t(471) = -2.11, p = .088); differences between control and consensus conditions were non-significant (b = -0.05, SE = 0.08, t(471) = -0.60, p = .822).


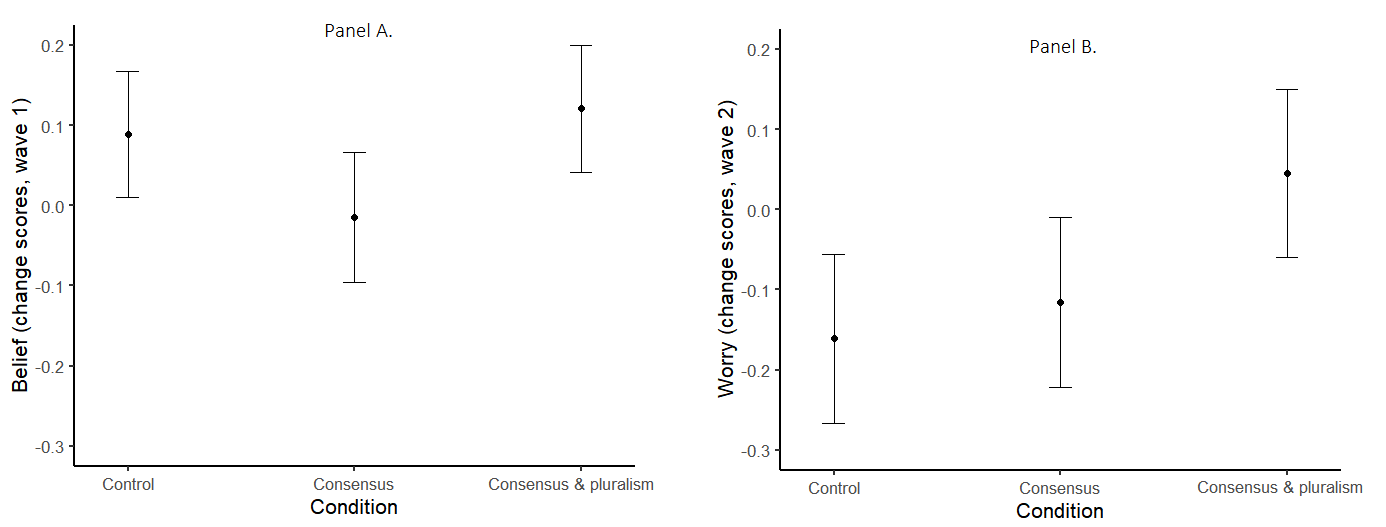


S2 Fig. The effects of consensus messaging on belief at wave 1 (Panel A) and on worry at wave 2 (Panel B).

S6b Table. *The effects of consensus messaging on vaccine belief, worry, and policy support in wave 1 and wave 2 in Study 2 (subjects with low consensus perception, low credibility of manipulation perception, and speeders are included; we do not control for demographic variables).*

|  | **Beliefs Wave 1** | | | **Beliefs Wave 2** | | | **Worry Wave 1** | | | **Worry Wave 2** | | | **Policy support Wave 1** | | | **Policy support Wave 2** | | |
| --- | --- | --- | --- | --- | --- | --- | --- | --- | --- | --- | --- | --- | --- | --- | --- | --- | --- | --- |
|  | *b* | *SE* | *p* | *b* | *SE* | *p* | *b* | *SE* | *p* | *b* | *SE* | *p* | *b* | *SE* | *p* | *b* | *SE* | *p* |
| Intercept | 0.09 | 0.04 | **0.010** | 0.04 | 0.05 | 0.440 | -0.06 | 0.04 | 0.095 | -0.16 | 0.05 | **0.002** | -0.03 | 0.02 | 0.157 | 0.07 | 0.04 | 0.071 |
| Condition [Consensus] | -0.06 | 0.05 | 0.204 | 0.08 | 0.07 | 0.217 | 0.06 | 0.05 | 0.224 | -0.01 | 0.07 | 0.921 | -0.01 | 0.03 | 0.661 | -0.01 | 0.05 | 0.798 |
| Condition [Consensus&pluralism] | 0.03 | 0.05 | 0.487 | 0.09 | 0.07 | 0.216 | -0.01 | 0.05 | 0.903 | 0.15 | 0.07 | **0.034** | 0.05 | 0.03 | 0.119 | -0.03 | 0.05 | 0.511 |
| Observations | 753 | | | 586 | | | 753 | | | 586 | | | 753 | | | 586 | | |
| R^2^ / R^2^ adjusted | 0.005 / 0.003 | | | 0.003 / 0.000 | | | 0.003 / 0.000 | | | 0.011 / 0.008 | | | 0.006 / 0.003 | | | 0.001 / -0.003 | | |

S7a Table. *The effects of consensus messaging and* ***ideology*** *on vaccine belief, worry, and policy support in wave 1 and wave 2 in Study 2*

|  | **Wave 1: Belief** | | | **Wave 2: Belief** | | | **Wave 1: Worry** | | | **Wave 2: Worry** | | | **Wave 1: Policy support** | | | **Wave 2: Policy support** | | |
| --- | --- | --- | --- | --- | --- | --- | --- | --- | --- | --- | --- | --- | --- | --- | --- | --- | --- | --- |
|  | *b* | *SE* | *p* | *b* | *SE* | *p* | *b* | *SE* | *p* | *b* | *SE* | *p* | *b* | *SE* | *p* | *b* | *SE* | *p* |
| Intercept | 0.13 | 0.07 | 0.053 | 0.10 | 0.10 | 0.321 | 0.03 | 0.07 | 0.692 | -0.19 | 0.11 | 0.074 | -0.04 | 0.05 | 0.348 | 0.11 | 0.08 | 0.181 |
| Age | -0.09 | 0.08 | 0.268 | -0.04 | 0.12 | 0.757 | -0.00 | 0.09 | 0.976 | -0.05 | 0.13 | 0.688 | 0.09 | 0.06 | 0.131 | -0.06 | 0.10 | 0.586 |
| Education | 0.01 | 0.07 | 0.932 | -0.08 | 0.10 | 0.391 | -0.12 | 0.08 | 0.125 | 0.08 | 0.11 | 0.456 | -0.03 | 0.05 | 0.521 | -0.05 | 0.08 | 0.590 |
| Gender | 0.01 | 0.04 | 0.784 | -0.01 | 0.06 | 0.880 | -0.02 | 0.05 | 0.661 | 0.01 | 0.06 | 0.880 | -0.01 | 0.03 | 0.696 | 0.03 | 0.05 | 0.595 |
| Condition [Consensus] | -0.11 | 0.05 | **0.030** | 0.01 | 0.07 | 0.943 | 0.04 | 0.06 | 0.425 | 0.05 | 0.08 | 0.520 | -0.01 | 0.04 | 0.783 | -0.04 | 0.06 | 0.467 |
| Condition [Consensus & pluralism] | 0.03 | 0.05 | 0.539 | 0.12 | 0.07 | 0.088 | 0.02 | 0.06 | 0.733 | 0.21 | 0.08 | **0.007** | 0.06 | 0.04 | 0.110 | -0.03 | 0.06 | 0.627 |
| Ideology | -0.09 | 0.15 | 0.527 | -0.02 | 0.21 | 0.917 | -0.14 | 0.16 | 0.387 | -0.08 | 0.23 | 0.715 | -0.10 | 0.10 | 0.308 | 0.10 | 0.18 | 0.584 |
| Ideology x Condition [Consensus] | -0.16 | 0.20 | 0.420 | -0.21 | 0.29 | 0.473 | 0.20 | 0.22 | 0.380 | 0.38 | 0.31 | 0.221 | 0.03 | 0.14 | 0.811 | -0.09 | 0.25 | 0.717 |
| Ideology x Condition [Consensus & pluralism] | 0.17 | 0.20 | 0.397 | -0.07 | 0.28 | 0.813 | 0.22 | 0.22 | 0.315 | 0.39 | 0.30 | 0.198 | 0.17 | 0.14 | 0.228 | -0.02 | 0.24 | 0.920 |
| Observations | 611 | | | 477 | | | 611 | | | 477 | | | 611 | | | 477 | | |
| R^2^ / R^2^ adjusted | 0.021 / 0.008 | | | 0.012 / -0.005 | | | 0.008 / -0.005 | | | 0.027 / 0.010 | | | 0.014 / 0.001 | | | 0.005 / -0.012 | | |

S7b Table. *The effects of consensus messaging and* ***ideology*** *on vaccine belief, worry, and policy support in wave 1 and wave 2 in Study 2 (subjects with low consensus perception, low credibility of manipulation perception, and speeders are included; we do not control for demographic variables).*

|  | **Wave 1: Belief** | | | **Wave 2: Belief** | | | **Wave 1: Worry** | | | **Wave 2: Worry** | | | **Wave 1: Policy support** | | | **Wave 2: Policy support** | | |
| --- | --- | --- | --- | --- | --- | --- | --- | --- | --- | --- | --- | --- | --- | --- | --- | --- | --- | --- |
|  | *b* | *SE* | *p* | *b* | *SE* | *p* | *b* | *SE* | *p* | *b* | *SE* | *p* | *b* | *SE* | *p* | *b* | *SE* | *p* |
| Intercept | 0.09 | 0.04 | **0.009** | 0.04 | 0.05 | 0.441 | -0.06 | 0.04 | 0.106 | -0.15 | 0.05 | **0.002** | -0.03 | 0.02 | 0.168 | 0.07 | 0.04 | 0.073 |
| Condition [Consensus] | -0.06 | 0.05 | 0.191 | 0.08 | 0.07 | 0.219 | 0.06 | 0.05 | 0.239 | -0.01 | 0.07 | 0.912 | -0.01 | 0.03 | 0.642 | -0.01 | 0.05 | 0.805 |
| Condition [Consensus&pluralism] | 0.03 | 0.05 | 0.491 | 0.08 | 0.07 | 0.223 | -0.01 | 0.05 | 0.861 | 0.15 | 0.07 | **0.031** | 0.05 | 0.03 | 0.123 | -0.03 | 0.05 | 0.526 |
| Ideology | -0.17 | 0.15 | 0.253 | 0.00 | 0.21 | 0.983 | -0.22 | 0.15 | 0.152 | -0.23 | 0.21 | 0.276 | -0.08 | 0.09 | 0.389 | 0.09 | 0.16 | 0.596 |
| Ideology x Condition [Consensus] | 0.09 | 0.20 | 0.664 | -0.12 | 0.28 | 0.670 | 0.30 | 0.21 | 0.162 | 0.38 | 0.29 | 0.184 | 0.04 | 0.12 | 0.768 | -0.02 | 0.21 | 0.931 |
| Ideology x Condition [Consensus&pluralism] | 0.37 | 0.19 | 0.059 | -0.08 | 0.27 | 0.775 | 0.10 | 0.21 | 0.613 | 0.46 | 0.28 | 0.106 | 0.13 | 0.12 | 0.281 | -0.04 | 0.21 | 0.861 |
| Observations | 753 | | | 586 | | | 753 | | | 586 | | | 753 | | | 586 | | |
| R^2^ / R^2^ adjusted | 0.011 / 0.004 | | | 0.004 / -0.004 | | | 0.007 / 0.000 | | | 0.017 / 0.008 | | | 0.008 / 0.001 | | | 0.002 / -0.007 | | |

S8a Table. *The effects of consensus messaging and* ***trust in scientists*** *on vaccine belief, worry, and policy support in wave 1 and wave 2 in Study 2.*

|  | **Wave 1: Belief** | | | **Wave 2: Belief** | | | **Wave 1: Worry** | | | **Wave 2: Worry** | | | **Wave 1: Policy support** | | | **Wave 2: Policy support** | | |
| --- | --- | --- | --- | --- | --- | --- | --- | --- | --- | --- | --- | --- | --- | --- | --- | --- | --- | --- |
|  | *b* | *SE* | *p* | *b* | *SE* | *p* | *b* | *SE* | *p* | *b* | *SE* | *p* | *b* | *SE* | *p* | *b* | *SE* | *p* |
| Intercept | 0.12 | 0.07 | 0.084 | 0.03 | 0.10 | 0.753 | 0.04 | 0.08 | 0.595 | -0.11 | 0.11 | 0.307 | -0.06 | 0.05 | 0.247 | 0.03 | 0.08 | 0.719 |
| Age | -0.09 | 0.08 | 0.304 | 0.01 | 0.12 | 0.949 | -0.01 | 0.09 | 0.929 | -0.12 | 0.13 | 0.347 | 0.10 | 0.06 | 0.104 | 0.00 | 0.10 | 0.984 |
| Education | 0.02 | 0.07 | 0.812 | -0.03 | 0.10 | 0.740 | -0.13 | 0.08 | 0.099 | 0.02 | 0.11 | 0.852 | -0.02 | 0.05 | 0.657 | 0.01 | 0.08 | 0.921 |
| Gender | 0.01 | 0.04 | 0.838 | 0.00 | 0.06 | 0.951 | -0.02 | 0.05 | 0.597 | -0.00 | 0.06 | 0.942 | -0.01 | 0.03 | 0.753 | 0.05 | 0.05 | 0.266 |
| Condition [Consensus] | -0.11 | 0.05 | **0.039** | 0.02 | 0.07 | 0.800 | 0.04 | 0.06 | 0.424 | 0.04 | 0.08 | 0.620 | -0.01 | 0.04 | 0.859 | -0.03 | 0.06 | 0.627 |
| Condition [Consensus & pluralism] | 0.03 | 0.05 | 0.523 | 0.13 | 0.07 | 0.071 | 0.01 | 0.05 | 0.804 | 0.20 | 0.08 | **0.008** | 0.06 | 0.04 | 0.099 | -0.01 | 0.06 | 0.805 |
| Trust | -0.01 | 0.14 | 0.943 | -0.30 | 0.19 | 0.128 | 0.14 | 0.15 | 0.367 | 0.13 | 0.21 | 0.519 | -0.00 | 0.10 | 0.971 | -0.44 | 0.16 | **0.007** |
| Trust x Condition [Consensus] | 0.10 | 0.19 | 0.590 | 0.23 | 0.27 | 0.397 | -0.37 | 0.21 | 0.078 | 0.07 | 0.29 | 0.814 | -0.03 | 0.13 | 0.819 | 0.12 | 0.23 | 0.583 |
| Trust x Condition [Consensus & pluralism] | 0.01 | 0.19 | 0.938 | 0.25 | 0.27 | 0.350 | 0.13 | 0.21 | 0.533 | -0.10 | 0.29 | 0.725 | -0.03 | 0.13 | 0.809 | 0.19 | 0.23 | 0.416 |
| Observations | 611 | | | 477 | | | 611 | | | 477 | | | 611 | | | 477 | | |
| R^2^ / R^2^ adjusted | 0.015 / 0.002 | | | 0.014 / -0.003 | | | 0.017 / 0.004 | | | 0.021 / 0.004 | | | 0.012 / -0.002 | | | - 1. 0.013 | | |

S8b Table. *The effects of consensus messaging and* ***trust in scientists*** *on vaccine belief, worry, and policy support in wave 1 and wave 2 in Study 2 (subjects with low consensus perception, low credibility of manipulation perception, and speeders are included; we do not control for demographic variables).*

|  | **Wave 1: Belief** | | | **Wave 2: Belief** | | | **Wave 1: Worry** | | | **Wave 2: Worry** | | | **Wave 1: Policy support** | | | **Wave 2: Policy support** | | |
| --- | --- | --- | --- | --- | --- | --- | --- | --- | --- | --- | --- | --- | --- | --- | --- | --- | --- | --- |
|  | *b* | *SE* | *p* | *b* | *SE* | *p* | *b* | *SE* | *p* | *b* | *SE* | *p* | *b* | *SE* | *p* | *b* | *SE* | *p* |
| Intercept | 0.09 | 0.04 | **0.010** | 0.04 | 0.05 | 0.422 | -0.06 | 0.04 | 0.093 | -0.16 | 0.05 | **0.002** | -0.03 | 0.02 | 0.158 | 0.07 | 0.04 | 0.062 |
| Condition [Consensus] | -0.06 | 0.05 | 0.192 | 0.08 | 0.07 | 0.245 | 0.06 | 0.05 | 0.238 | -0.00 | 0.07 | 0.954 | -0.01 | 0.03 | 0.661 | -0.02 | 0.05 | 0.734 |
| Condition [Consensus&pluralism] | 0.03 | 0.05 | 0.495 | 0.08 | 0.07 | 0.229 | -0.01 | 0.05 | 0.851 | 0.15 | 0.07 | **0.039** | 0.05 | 0.03 | 0.121 | -0.03 | 0.05 | 0.544 |
| Trust | 0.12 | 0.12 | 0.353 | -0.17 | 0.17 | 0.331 | 0.13 | 0.13 | 0.312 | 0.08 | 0.18 | 0.657 | 0.02 | 0.08 | 0.825 | -0.26 | 0.13 | 0.056 |
| Trust x Condition [Consensus] | -0.25 | 0.17 | 0.133 | -0.28 | 0.23 | 0.230 | -0.30 | 0.18 | 0.090 | 0.17 | 0.24 | 0.476 | -0.02 | 0.10 | 0.853 | 0.01 | 0.18 | 0.971 |
| Trust x Condition [Consensus&pluralism] | -0.06 | 0.17 | 0.711 | 0.21 | 0.24 | 0.370 | 0.14 | 0.18 | 0.428 | 0.05 | 0.25 | 0.831 | -0.01 | 0.11 | 0.930 | 0.10 | 0.18 | 0.581 |
| Observations | 753 | | | 586 | | | 753 | | | 586 | | | 753 | | | 586 | | |
| R^2^ / R^2^ adjusted | 0.009 / 0.002 | | | 0.020 / 0.011 | | | 0.013 / 0.007 | | | 0.017 / 0.008 | | | 0.006 / -0.000 | | | 0.017 / 0.009 | | |

## **S2.2. Testing hypotheses with SEM**

S9 Table. *Fit of SEM models in Study 3*

|  | Wave 1 | | | | | | | Wave 2 | | | | | |
| --- | --- | --- | --- | --- | --- | --- | --- | --- | --- | --- | --- | --- | --- |
|  | Control vs. Consensus | | | Control vs. Consensus & pluralism | | Consensus vs. Consensus & pluralism | | Control vs. Consensus | | Control vs. Consensus & pluralism | | Consensus vs. Consensus & pluralism | |
| χ^2^(4) | | 10.31 | 8.84 | | 17.50 | | 5.40 | | 13.54 | | 14.31 | |  |
| p | | 0.035 | 0.065 | | 0.002 | | 0.249 | | 0.009 | | 0.006 | |  |
| CFI | | 0.90 | 0.89 | | 0.28 | | 0.97 | | 0.74 | | 0.74 | |  |
| SRMR | | 0.04 | 0.04 | | 0.05 | | 0.03 | | 0.05 | | 0.05 | |  |
| RMSEA | | 0.05 | 0.05 | | 0.09 | | 0.03 | | 0.09 | | 0.09 | |  |
| 95% CI RMSEA | | (0.01-0.09) | (0.00 – 0.10) | | (0.05 – 0.14) | | (0.00 – 0.10) | | (0.04 – 0.14) | | - 1. – 0.14) | |  |

### S2.2.1. Wave 1

S10 Table. *Path coefficients and indirect effects for comparison between control vs. consensus conditions at wave 1 in Study 2*

|  |  |  |  |  |  |  |  | 95% CI | |
| --- | --- | --- | --- | --- | --- | --- | --- | --- | --- |
| Paths |  |  |  | *b* | SE | *z* | *p* | Lower | Upper |
| Consensus messaging | → | Perceived scientific consensus |  | 9.320 | 1.163 | 8.017 | **<.001** | 7.072 | 11.656 |
| Perceived scientific consensus | → | Belief |  | 0.000 | 0.002 | -0.083 | .934 | -0.003 | 0.003 |
| Perceived scientific consensus | → | Worry |  | 0.000 | 0.002 | -0.177 | .860 | -0.004 | 0.003 |
| Belief | → | Worry |  | -0.107 | 0.066 | -1.612 | .107 | -0.234 | 0.030 |
| Belief | → | Policy support |  | 0.048 | 0.032 | 1.505 | .132 | -0.013 | 0.111 |
| Worry | → | Policy support |  | 0.036 | 0.034 | 1.054 | .292 | -0.033 | 0.104 |
| ***Indirect effects of perceived consensus*** |  |  |  |  |  |  |  |  |  |
| Through belief |  |  |  | 0.000 | 0.000 | -0.068 | .946 | 0.000 | 0.000 |
| Through worry |  |  |  | 0.000 | 0.000 | -0.124 | .901 | 0.000 | 0.000 |
| Through belief and worry |  |  |  | 0.000 | 0.000 | 0.050 | .960 | 0.000 | 0.000 |
| Combined indirect effect |  |  |  | 0.000 | 0.000 | -0.132 | .895 | 0.000 | 0.000 |
| ***Indirect effects of consensus messaging*** |  |  |  |  |  |  |  |  |  |
| Through perceived consensus and belief |  |  |  | 0.000 | 0.001 | -0.068 | .946 | -0.002 | 0.002 |
| Through perceived consensus and worry |  |  |  | 0.000 | 0.001 | -0.123 | .902 | -0.003 | 0.001 |
| Through perceived consensus, belief, and worry |  |  |  | 0.000 | 0.000 | 0.050 | .960 | 0.000 | 0.000 |
| Combined indirect effect |  |  |  | 0.000 | 0.001 | -0.130 | .896 | -0.003 | 0.002 |

S11 Table. *Path coefficients and indirect effects for comparison between control vs. consensus & pluralism conditions at wave 1 in Study 2*

|  |  |  |  |  |  |  |  | 95% CI | |
| --- | --- | --- | --- | --- | --- | --- | --- | --- | --- |
| Paths |  |  |  | *b* | SE | *z* | *p* | Lower | Upper |
| Consensus messaging | → | Perceived scientific consensus |  | 8.862 | 1.384 | 6.405 | **<.001** | 6.226 | 11.591 |
| Perceived scientific consensus | → | Belief |  | 0.002 | 0.002 | 1.025 | .305 | -0.001 | 0.006 |
| Perceived scientific consensus | → | Worry |  | 0.001 | 0.003 | 0.453 | .650 | -0.005 | 0.006 |
| Belief | → | Worry |  | -0.080 | 0.093 | -0.854 | .393 | -0.247 | 0.119 |
| Belief | → | Policy support |  | 0.036 | 0.051 | 0.707 | .480 | -0.055 | 0.148 |
| Worry | → | Policy support |  | 0.032 | 0.046 | 0.687 | .492 | -0.059 | 0.123 |
| ***Indirect effects of perceived consensus*** |  |  |  |  |  |  |  |  |  |
| Through belief |  |  |  | 0.000 | 0.000 | 0.420 | .674 | 0.000 | 0.001 |
| Through worry |  |  |  | 0.000 | 0.000 | 0.221 | .825 | 0.000 | 0.001 |
| Through belief and worry |  |  |  | 0.000 | 0.000 | -0.292 | .770 | 0.000 | 0.000 |
| Combined indirect effect |  |  |  | 0.000 | 0.000 | 0.430 | .667 | 0.000 | 0.001 |
| ***Indirect effects of consensus messaging*** |  |  |  |  |  |  |  |  |  |
| Through perceived consensus and belief |  |  |  | 0.001 | 0.001 | 0.419 | .676 | -0.001 | 0.006 |
| Through perceived consensus and worry |  |  |  | 0.000 | 0.002 | 0.217 | .829 | -0.001 | 0.007 |
| Through perceived consensus, belief, and worry |  |  |  | 0.000 | 0.000 | -0.289 | .773 | -0.001 | 0.000 |
| Combined indirect effect |  |  |  | 0.001 | 0.002 | 0.420 | .674 | -0.002 | 0.007 |

S12 Table. *Path coefficients and indirect effects for comparison between consensus vs. consensus & pluralism conditions at wave 1 in Study 2*

|  |  |  |  |  |  |  |  | 95% CI | |
| --- | --- | --- | --- | --- | --- | --- | --- | --- | --- |
| Paths |  |  | label | *b* | SE | *z* | *p* | Lower | Upper |
| Consensus messaging | → | Perceived scientific consensus |  | -0.932 | 1.609 | -0.579 | .563 | -4.115 | 2.128 |
| Perceived scientific consensus | → | Belief |  | 0.000 | 0.002 | 0.163 | .871 | -0.004 | 0.005 |
| Perceived scientific consensus | → | Worry |  | -0.002 | 0.002 | -0.873 | .383 | -0.007 | 0.002 |
| Belief | → | Worry |  | -0.095 | 0.076 | -1.239 | .215 | -0.243 | 0.053 |
| Belief | → | Policy support |  | 0.071 | 0.038 | 1.865 | .062 | 0.003 | 0.152 |
| Worry | → | Policy support |  | 0.046 | 0.041 | 1.130 | .258 | -0.034 | 0.129 |
| ***Indirect effects of perceived consensus*** |  |  |  |  |  |  |  |  |  |
| Through belief |  |  |  | 0.000 | 0.000 | 0.141 | .888 | 0.000 | 0.000 |
| Through worry |  |  |  | 0.000 | 0.000 | -0.582 | .560 | -0.001 | 0.000 |
| Through belief and worry |  |  |  | 0.000 | 0.000 | -0.098 | .922 | 0.000 | 0.000 |
| Combined indirect effect |  |  |  | 0.000 | 0.000 | -0.320 | .749 | -0.001 | 0.000 |
| ***Indirect effects of consensus messaging*** |  |  |  |  |  |  |  |  |  |
| Through perceived consensus and belief |  |  |  | 0.000 | 0.000 | -0.067 | .947 | -0.001 | 0.000 |
| Through perceived consensus and worry |  |  |  | 0.000 | 0.000 | 0.255 | .799 | 0.000 | 0.002 |
| Through perceived consensus, belief, and worry |  |  |  | 0.000 | 0.000 | 0.050 | .960 | 0.000 | 0.000 |
| Combined indirect effect |  |  |  | 0.000 | 0.000 | 0.150 | .881 | 0.000 | 0.002 |

### S2.2.2. Wave 2

S13 Table. *Path coefficients and indirect effects for comparison between control vs. consensus conditions at wave 2 in Study 2*

|  |  |  |  |  |  |  |  | 95% CI | |
| --- | --- | --- | --- | --- | --- | --- | --- | --- | --- |
| Paths |  |  | label | *b* | SE | *z* | *p* | Lower | Upper |
| Consensus messaging | → | Perceived scientific consensus |  | 6.956 | 1.731 | 4.018 | **<.001** | 3.707 | 10.373 |
| Perceived scientific consensus | → | Belief |  | 0.002 | 0.002 | 0.746 | .456 | -0.003 | 0.007 |
| Perceived scientific consensus | → | Worry |  | -0.005 | 0.003 | -1.879 | .060 | -0.011 | 0.000 |
| Belief | → | Worry |  | -0.141 | 0.088 | -1.605 | .109 | -0.343 | 0.004 |
| Belief | → | Policy support |  | 0.218 | 0.058 | 3.796 | **<.001** | 0.116 | 0.340 |
| Worry | → | Policy support |  | 0.003 | 0.047 | 0.064 | .949 | -0.085 | 0.100 |
| ***Indirect effects of perceived consensus*** |  |  |  |  |  |  |  |  |  |
| Through belief |  |  |  | 0.000 | 0.001 | 0.717 | .474 | -0.001 | 0.002 |
| Through worry |  |  |  | 0.000 | 0.000 | -0.061 | .952 | -0.001 | 0.000 |
| Through belief and worry |  |  |  | 0.000 | 0.000 | -0.029 | .977 | 0.000 | 0.000 |
| Combined indirect effect |  |  |  | 0.000 | 0.001 | 0.645 | .519 | -0.001 | 0.002 |
| ***Indirect effects of consensus messaging*** |  |  |  |  |  |  |  |  |  |
| Through perceived consensus and belief |  |  |  | 0.003 | 0.004 | 0.714 | .475 | -0.004 | 0.012 |
| Through perceived consensus and worry |  |  |  | 0.000 | 0.002 | -0.059 | .953 | -0.005 | 0.003 |
| Through perceived consensus, belief, and worry |  |  |  | 0.000 | 0.000 | -0.029 | .977 | -0.001 | 0.000 |
| Combined indirect effect |  |  |  | 0.003 | 0.004 | 0.645 | .519 | -0.006 | 0.011 |

S14 Table. *Path coefficients and indirect effects for comparison between control vs. consensus & pluralism conditions at wave 2 in Study 2*

|  |  |  |  |  |  |  |  | 95% CI | |
| --- | --- | --- | --- | --- | --- | --- | --- | --- | --- |
| Paths |  |  | label | *b* | SE | *z* | *p* | Lower | Upper |
| Consensus messaging | → | Perceived scientific consensus |  | 5.087 | 1.501 | 3.390 | .001 | 2.210 | 8.076 |
| Perceived scientific consensus | → | Belief |  | 0.002 | 0.002 | 0.885 | .376 | -0.002 | 0.007 |
| Perceived scientific consensus | → | Worry |  | 0.005 | 0.003 | 1.626 | .104 | -0.001 | 0.011 |
| Belief | → | Worry |  | -0.052 | 0.076 | -0.690 | .490 | -0.220 | 0.085 |
| Belief | → | Policy support |  | 0.223 | 0.054 | 4.110 | <.001 | 0.129 | 0.340 |
| Worry | → | Policy support |  | -0.018 | 0.054 | -0.338 | .735 | -0.118 | 0.094 |
| ***Indirect effects of perceived consensus*** |  |  |  |  |  |  |  |  |  |
| Through belief |  |  |  | 0.000 | 0.001 | 0.830 | .407 | 0.000 | 0.002 |
| Through worry |  |  |  | 0.000 | 0.000 | -0.284 | .776 | -0.001 | 0.000 |
| Through belief and worry |  |  |  | 0.000 | 0.000 | 0.139 | .890 | 0.000 | 0.000 |
| Combined indirect effect |  |  |  | 0.000 | 0.001 | 0.589 | .556 | -0.001 | 0.002 |
| ***Indirect effects of consensus messaging*** |  |  |  |  |  |  |  |  |  |
| Through perceived consensus and belief |  |  |  | 0.002 | 0.003 | 0.769 | .442 | -0.002 | 0.012 |
| Through perceived consensus and worry |  |  |  | 0.000 | 0.002 | -0.267 | .789 | -0.005 | 0.002 |
| Through perceived consensus, belief, and worry |  |  |  | 0.000 | 0.000 | 0.132 | .895 | 0.000 | 0.000 |
| Combined indirect effect |  |  |  | 0.002 | 0.004 | 0.545 | .585 | -0.004 | 0.012 |

S15 Table. *Path coefficients and indirect effects for comparison between consensus vs. consensus & pluralism conditions at wave 2 in Study 2*

|  |  |  |  |  |  |  |  | 95% CI | |
| --- | --- | --- | --- | --- | --- | --- | --- | --- | --- |
| Paths |  |  | label | *b* | SE | *z* | *p* | Lower | Upper |
| Consensus messaging | → | Perceived scientific consensus |  | -1.869 | 1.778 | -1.051 | .293 | -5.373 | 1.641 |
| Perceived scientific consensus | → | Belief |  | 0.003 | 0.003 | 0.883 | .377 | -0.003 | 0.008 |
| Perceived scientific consensus | → | Worry |  | -0.005 | 0.003 | -1.714 | .086 | -0.011 | 0.001 |
| Belief | → | Worry |  | -0.143 | 0.097 | -1.467 | .142 | -0.355 | 0.026 |
| Belief | → | Policy support |  | 0.190 | 0.054 | 3.508 | <.001 | 0.094 | 0.308 |
| Worry | → | Policy support |  | -0.007 | 0.041 | -0.168 | .867 | -0.087 | 0.076 |
| ***Indirect effects of perceived consensus*** |  |  |  |  |  |  |  |  |  |
| Through belief |  |  |  | 0.000 | 0.001 | 0.810 | .418 | 0.000 | 0.002 |
| Through worry |  |  |  | 0.000 | 0.000 | 0.160 | .873 | 0.000 | 0.001 |
| Through belief and worry |  |  |  | 0.000 | 0.000 | 0.095 | .925 | 0.000 | 0.000 |
| Combined indirect effect |  |  |  | 0.001 | 0.001 | 0.864 | .388 | -0.001 | 0.002 |
| ***Indirect effects of consensus messaging*** |  |  |  |  |  |  |  |  |  |
| Through perceived consensus and belief |  |  |  | -0.001 | 0.002 | -0.544 | .587 | -0.007 | 0.001 |
| Through perceived consensus and worry |  |  |  | 0.000 | 0.001 | -0.110 | .913 | -0.002 | 0.001 |
| Through perceived consensus, belief, and worry |  |  |  | 0.000 | 0.000 | -0.073 | .942 | 0.000 | 0.000 |
| Combined indirect effect |  |  |  | -0.001 | 0.002 | -0.562 | .574 | -0.007 | 0.001 |

# **S3. Study 3**

## S3.1. Analysis of sensitization effects

S16a Table. *The effects of consensus messaging and study design manipulations on perceived consensus and vaccine attitudes and intentions in Study 3*

| DVs: | Perception of consensus | | | Worry | | | Belief | | | Policy support | | | Vaccination intention (unvaccinated) | | | Booster shot intention (vaccinated) | | |
| --- | --- | --- | --- | --- | --- | --- | --- | --- | --- | --- | --- | --- | --- | --- | --- | --- | --- | --- |
|  | *b* | *SE* | *p* | *b* | *SE* | *p* | *b* | *SE* | *p* | *b* | *SE* | *p* | *b* | *SE* | *p* | *b* | *SE* | *p* |
| Intercept | 56.50 | 2.60 | <0.001 | 3.76 | 0.13 | <0.001 | 2.39 | 0.12 | <0.001 | 1.78 | 0.15 | <0.001 | 1.62 | 0.16 | <0.001 | 2.43 | 0.17 | <0.001 |
| Age | 14.83 | 3.74 | <0.001 | -1.79 | 0.19 | <0.001 | 1.92 | 0.18 | <0.001 | 2.63 | 0.21 | <0.001 | 0.05 | 0.26 | 0.852 | 2.59 | 0.24 | <0.001 |
| Gender | -4.33 | 1.49 | 0.004 | 0.20 | 0.08 | 0.007 | -0.12 | 0.07 | 0.104 | -0.23 | 0.08 | 0.007 | -0.05 | 0.10 | 0.633 | -0.22 | 0.09 | 0.021 |
| Education | 14.63 | 2.48 | <0.001 | -0.64 | 0.13 | <0.001 | 0.59 | 0.12 | <0.001 | 0.54 | 0.14 | <0.001 | -0.15 | 0.18 | 0.413 | 0.54 | 0.15 | 0.001 |
| Design [Post-only] | 0.44 | 2.07 | 0.832 | -0.07 | 0.10 | 0.475 | -0.00 | 0.10 | 0.983 | 0.03 | 0.12 | 0.780 | 0.06 | 0.14 | 0.657 | -0.07 | 0.13 | 0.616 |
| Message [Consensus] | 10.05 | 2.10 | <0.001 | -0.09 | 0.11 | 0.371 | 0.09 | 0.10 | 0.347 | 0.03 | 0.12 | 0.771 | 0.03 | 0.14 | 0.816 | 0.06 | 0.13 | 0.666 |
| Design x Message | 0.07 | 2.92 | 0.982 | 0.17 | 0.15 | 0.239 | -0.19 | 0.14 | 0.181 | -0.12 | 0.16 | 0.443 | 0.07 | 0.19 | 0.711 | 0.08 | 0.18 | 0.655 |
| Observations | 1051 | | | 1051 | | | 1051 | | | 1051 | | | 328 | | | 723 | | |
| R^2^ / R^2^ adjusted | 0.083 / 0.078 | | | 0.093 / 0.088 | | | 0.108 / 0.103 | | | 0.139 / 0.134 | | | 0.010 / -0.009 | | | 0.150 / 0.143 | | |

*Note.* Responses to post-measurements were used as DVs (i.e., not the change scores). Vaccination intention was measured among unvaccinated and receiving a booster shot intention (if recommended by experts) among people who reported to have received at least one dose of vaccine.

S16b Table. *The effects of consensus messaging and study design manipulations on perceived consensus and vaccine attitudes and intentions in Study 3 (subjects with low consensus perception and speeders are included; we do not control for demographic variables).*

|  | Perception of consensus | | | Worry | | | Belief | | | Policy support | | | Vaccination int. (unvaxxed) | | | Boosting int. (vaxxed) | | |
| --- | --- | --- | --- | --- | --- | --- | --- | --- | --- | --- | --- | --- | --- | --- | --- | --- | --- | --- |
|  | *b* | *SE* | *p* | *b* | *SE* | *p* | *b* | *SE* | *p* | *b* | *SE* | *p* | *b* | *SE* | *p* | *b* | *SE* | *p* |
| Intercept | 66.43 | 1.56 | <0.001 | 2.91 | 0.08 | <0.001 | 3.29 | 0.08 | <0.001 | 2.84 | 0.09 | <0.001 | 1.52 | 0.09 | <0.001 | 3.58 | 0.10 | <0.001 |
| Design [Post-only] | 0.69 | 2.17 | 0.751 | -0.10 | 0.11 | 0.337 | 0.04 | 0.11 | 0.687 | 0.08 | 0.12 | 0.505 | 0.05 | 0.13 | 0.707 | -0.01 | 0.14 | 0.916 |
| Message [Consensus] | 11.06 | 2.19 | <0.001 | -0.09 | 0.11 | 0.427 | 0.09 | 0.11 | 0.413 | 0.03 | 0.12 | 0.808 | 0.02 | 0.13 | 0.887 | 0.05 | 0.14 | 0.747 |
| Design x Message conditions | -0.29 | 3.06 | 0.925 | 0.13 | 0.15 | 0.402 | -0.16 | 0.15 | 0.290 | -0.07 | 0.17 | 0.678 | 0.15 | 0.19 | 0.430 | 0.14 | 0.20 | 0.495 |
| Observations | 1073 | | | 1073 | | | 1073 | | | 1073 | | | 342 | | | 731 | | |
| R^2^ / R^2^ adjusted | 0.046 / 0.043 | | | 0.001 / -0.002 | | | 0.001 / -0.002 | | | 0.000 / -0.002 | | | 0.011 / 0.002 | | | 0.003 / -0.001 | | |

## S3.2. Testing hypotheses with OLS regression

### **S3.2.1. The effects of consensus messaging on perception of scientific consensus**

S17a Table. *The effects of consensus messaging and moderators on perceived scientific consensus in the “pre-post” condition (change scores) and the “post-only” condition in Study 3.*

|  | Pre-post | | | Post-only | | | Pre-post: Ideology | | | Post-only: Ideology | | | Pre-post: Trust | | | Post-only: Trust | | | Pre-post: Priors | | |
| --- | --- | --- | --- | --- | --- | --- | --- | --- | --- | --- | --- | --- | --- | --- | --- | --- | --- | --- | --- | --- | --- |
|  | *b* | *SE* | *p* | *b* | *SE* | *p* | *b* | *SE* | *p* | *b* | *SE* | *p* | *b* | *SE* | *p* | *b* | *SE* | *p* | *b* | *SE* | *p* |
| Intercept | 3.04 | 2.79 | 0.276 | 57.27 | 3.10 | <0.001 | 3.09 | 2.83 | 0.275 | 61.52 | 3.07 | <0.001 | 1.96 | 2.80 | 0.483 | 63.01 | 2.79 | <0.001 | -0.66 | 2.67 | 0.806 |
| Age | -5.07 | 4.42 | 0.252 | 12.43 | 4.83 | 0.010 | -4.97 | 4.46 | 0.265 | 8.28 | 4.71 | 0.079 | -2.57 | 4.48 | 0.566 | 1.76 | 4.36 | 0.686 | 1.33 | 4.23 | 0.753 |
| Gender | 2.67 | 1.73 | 0.124 | -6.60 | 1.96 | 0.001 | 2.66 | 1.74 | 0.126 | -7.17 | 1.89 | <0.001 | 2.35 | 1.72 | 0.172 | -3.67 | 1.75 | 0.036 | 1.48 | 1.64 | 0.367 |
| Education | -2.90 | 2.93 | 0.323 | 17.59 | 3.21 | <0.001 | -3.03 | 2.98 | 0.309 | 13.21 | 3.17 | <0.001 | -2.20 | 2.92 | 0.450 | 11.20 | 2.89 | <0.001 | 0.86 | 2.80 | 0.759 |
| Message [Consensus] | 10.24 | 1.70 | <0.001 | 10.27 | 1.91 | <0.001 | 10.18 | 1.70 | <0.001 | 10.92 | 1.84 | <0.001 | 10.59 | 1.68 | <0.001 | 11.21 | 1.69 | <0.001 | 10.20 | 1.60 | <0.001 |
| Ideology |  |  |  |  |  |  | 1.84 | 5.52 | 0.740 | -27.92 | 6.11 | <0.001 |  |  |  |  |  |  |  |  |  |
| Ideology x Message |  |  |  |  |  |  | -4.80 | 7.80 | 0.539 | 0.32 | 8.36 | 0.970 |  |  |  |  |  |  |  |  |  |
| Trust in scientists |  |  |  |  |  |  |  |  |  |  |  |  | 2.68 | 4.64 | 0.564 | 47.36 | 4.57 | <0.001 |  |  |  |
| Trust x Message |  |  |  |  |  |  |  |  |  |  |  |  | -19.32 | 6.32 | 0.002 | -17.76 | 6.17 | 0.004 |  |  |  |
| Priors |  |  |  |  |  |  |  |  |  |  |  |  |  |  |  |  |  |  | -18.80 | 4.72 | <0.001 |
| Priors x Message |  |  |  |  |  |  |  |  |  |  |  |  |  |  |  |  |  |  | -14.94 | 6.54 | 0.023 |
| Observations | 508 | | | 543 | | | 508 | | | 543 | | | 508 | | | 543 | | | 508 | | |
| R^2^ / R^2^ adjusted | 0.074 / 0.067 | | | 0.110 / 0.103 | | | 0.075 / 0.064 | | | 0.174 / 0.164 | | | 0.100 / 0.089 | | | 0.305 / 0.297 | | | 0.183 / 0.173 | | |

*Moderation of the consensus messaging by priors*

As shown in S3 Fig, people with low priors as compared to people with high priors updated their perceived consensus more in the consensus messaging condition (b = 16.19, SE = 2.24, t(501) = 7.25, p < .001); quite surprisingly, in the control condition, people with low priors also updated their belief more than people with high priors but this difference could not be attributed to the consensus messaging (b = 9.02, SE = 2.26, t(501) = 3.99, p < .001).


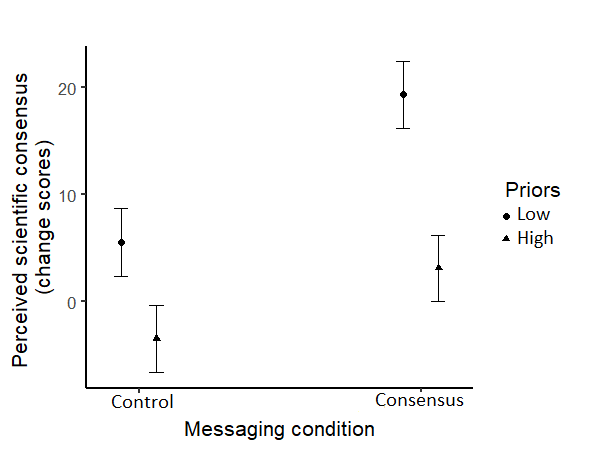


S3 Fig. The effects of consensus messaging and prior perception of consensus on perceived consensus in Study 3 (in the pre-post design condition).

S17b Table. *The effects of consensus messaging and moderators on perceived scientific consensus in the “pre-post” condition (change scores) and the “post-only” condition in Study 3 (subjects with low consensus perception and speeders are included; we do not control for demographic variables).*

|  | Pre-post | | | Post-only | | | Pre-post: Ideology | | | Post-only: Ideology | | | Pre-post: Trust | | | Post-only: Trust | | | Pre-post: Priors | | |  |
| --- | --- | --- | --- | --- | --- | --- | --- | --- | --- | --- | --- | --- | --- | --- | --- | --- | --- | --- | --- | --- | --- | --- |
|  | *b* | *SE* | *p* | *b* | *SE* | *p* | *b* | *SE* | *p* | *b* | *SE* | *p* | *b* | *SE* | *p* | *b* | *SE* | *p* | *b* | *SE* | *p* |  |
| Intercept | 1.10 | 1.25 | 0.378 | 67.12 | 1.44 | <0.001 | 1.10 | 1.25 | 0.380 | 67.06 | 1.38 | <0.001 | 1.10 | 1.22 | 0.366 | 66.91 | 1.26 | <0.001 | 1.11 | 1.15 | 0.334 | |
| Message [Consensus] | 11.16 | 1.75 | <0.001 | 10.77 | 2.04 | <0.001 | 11.17 | 1.75 | <0.001 | 11.43 | 1.95 | <0.001 | 11.60 | 1.71 | <0.001 | 11.58 | 1.78 | <0.001 | 11.13 | 1.61 | <0.001 | |
| Ideology |  |  |  |  |  |  | 3.03 | 5.59 | 0.589 | -33.19 | 6.36 | <0.001 |  |  |  |  |  |  |  |  |  | |
| Ideology x Message |  |  |  |  |  |  | -2.72 | 7.99 | 0.734 | 0.46 | 8.86 | 0.959 |  |  |  |  |  |  |  |  |  | |
| Trust |  |  |  |  |  |  |  |  |  |  |  |  | 0.76 | 4.53 | 0.868 | 53.49 | 4.65 | <0.001 |  |  |  | |
| Trust x Message |  |  |  |  |  |  |  |  |  |  |  |  | -23.23 | 6.33 | <0.001 | -23.20 | 6.47 | <0.001 |  |  |  | |
| Priors |  |  |  |  |  |  |  |  |  |  |  |  |  |  |  |  |  |  | -17.46 | 4.48 | <0.001 | |
| Priors x Message |  |  |  |  |  |  |  |  |  |  |  |  |  |  |  |  |  |  | -21.95 | 6.29 | 0.001 | |
| Observations | 522 | | | 551 | | | 522 | | | 551 | | | 522 | | | 551 | | | 522 | | |  |
| R^2^ / R^2^ adjusted | 0.073 / 0.071 | | | 0.048 / 0.046 | | | 0.073 / 0.068 | | | 0.136 / 0.131 | | | 0.117 / 0.112 | | | 0.281 / 0.278 | | | 0.216 / 0.212 | | |  |

### **S3.2.2. The effects of consensus messaging on vaccine attitudes and intentions**

S18a Table. *The effects of consensus messaging on vaccine worry, belief, and policy support in Study 3*

|  | **Pre-post: Worry** | | | **Post-only: Worry** | | | **Pre-post: Beliefs** | | | **Post-only: Beliefs** | | | **Pre-post: Policy support** | | | **Post-only: Policy support** | | |
| --- | --- | --- | --- | --- | --- | --- | --- | --- | --- | --- | --- | --- | --- | --- | --- | --- | --- | --- |
|  | *b* | *SE* | *p* | *b* | *SE* | *p* | *b* | *SE* | *p* | *b* | *SE* | *p* | *b* | *SE* | *p* | *b* | *SE* | *p* |
| Intercept | -0.06 | 0.07 | 0.388 | 3.69 | 0.16 | **<0.001** | -0.04 | 0.08 | 0.608 | 2.43 | 0.15 | **<0.001** | -0.07 | 0.05 | 0.124 | 1.75 | 0.18 | **<0.001** |
| Age | 0.13 | 0.11 | 0.254 | -1.78 | 0.25 | **<0.001** | 0.04 | 0.13 | 0.760 | 1.90 | 0.24 | **<0.001** | 0.11 | 0.07 | 0.117 | 2.65 | 0.28 | **<0.001** |
| Gender | -0.07 | 0.04 | 0.130 | 0.32 | 0.10 | **0.002** | 0.07 | 0.05 | 0.165 | -0.19 | 0.10 | **0.045** | -0.02 | 0.03 | 0.457 | -0.31 | 0.11 | **0.006** |
| Education | 0.09 | 0.08 | 0.249 | -0.77 | 0.17 | **<0.001** | 0.01 | 0.09 | 0.931 | 0.61 | 0.16 | **<0.001** | 0.03 | 0.05 | 0.564 | 0.73 | 0.18 | **<0.001** |
| Message [Consensus] | -0.00 | 0.04 | 0.928 | 0.07 | 0.10 | 0.466 | -0.01 | 0.05 | 0.920 | -0.09 | 0.09 | 0.347 | -0.01 | 0.03 | 0.835 | -0.09 | 0.11 | 0.431 |
| Observations | 508 | | | 543 | | | 508 | | | 543 | | | 508 | | | 543 | | |
| R^2^ / R^2^ adjusted | 0.008 / -0.000 | | | 0.114 / 0.107 | | | 0.004 / -0.004 | | | 0.119 / 0.113 | | | 0.006 / -0.002 | | | 0.161 / 0.155 | | |

S18b Table. *The effects of consensus messaging on vaccine worry, belief, and policy support in Study 3 (subjects with low consensus perception and speeders are included; we do not control for demographic variables).*

|  | Pre-post: Worry | | | Post-only: Worry | | | Pre-post: Beliefs | | | Post-only: Beliefs | | | Pre-post: Policy support | | | Post-only: Policy support | | |
| --- | --- | --- | --- | --- | --- | --- | --- | --- | --- | --- | --- | --- | --- | --- | --- | --- | --- | --- |
|  | *b* | *SE* | *p* | *b* | *SE* | *p* | *b* | *SE* | *p* | *b* | *SE* | *p* | *b* | *SE* | *p* | *b* | *SE* | *p* |
| Intercept | -0.02 | 0.03 | 0.618 | 2.81 | 0.07 | <0.001 | 0.02 | 0.04 | 0.662 | 3.33 | 0.07 | <0.001 | -0.03 | 0.02 | 0.145 | 2.92 | 0.08 | <0.001 |
| Message [Consensus] | 0.01 | 0.04 | 0.754 | 0.04 | 0.10 | 0.693 | -0.01 | 0.05 | 0.901 | -0.07 | 0.10 | 0.484 | -0.00 | 0.03 | 0.854 | -0.04 | 0.12 | 0.724 |
| Observations | 522 | | | 551 | | | 522 | | | 551 | | | 522 | | | 551 | | |
| R^2^ / R^2^ adjusted | 0.000 / -0.002 | | | 0.000 / -0.002 | | | 0.000 / -0.002 | | | 0.001 / -0.001 | | | 0.000 / -0.002 | | | 0.000 / -0.002 | | |

S19a Table. *The effects of consensus messaging on COVID-19 vaccination intention in Study 3*

|  | Pre-post: Vax. int. | | | Post-only: Vax. int. | | | Pre-post: Boost. int. | | | Post-only: Boost. int. | | |
| --- | --- | --- | --- | --- | --- | --- | --- | --- | --- | --- | --- | --- |
|  | *b* | *SE* | *p* | *b* | *SE* | *p* | *b* | *SE* | *p* | *b* | *SE* | *p* |
| Intercept | -0.02 | 0.07 | 0.797 | 1.57 | 0.23 | <0.001 | -0.06 | 0.11 | 0.548 | 2.38 | 0.22 | <0.001 |
| Age | -0.07 | 0.12 | 0.546 | 0.31 | 0.39 | 0.433 | 0.21 | 0.16 | 0.179 | 2.31 | 0.32 | <0.001 |
| Gender | -0.01 | 0.05 | 0.898 | -0.09 | 0.14 | 0.511 | -0.00 | 0.06 | 0.965 | -0.19 | 0.13 | 0.161 |
| Education | 0.03 | 0.08 | 0.721 | -0.01 | 0.26 | 0.965 | -0.05 | 0.10 | 0.644 | 0.67 | 0.21 | 0.002 |
| Message [Consensus] | 0.05 | 0.04 | 0.243 | 0.10 | 0.14 | 0.485 | -0.06 | 0.06 | 0.357 | 0.14 | 0.13 | 0.273 |
| Observations | 160 | | | 168 | | | 348 | | | 375 | | |
| R^2^ / R^2^ adjusted | 0.013 / -0.013 | | | 0.011 / -0.014 | | | 0.010 / -0.002 | | | - 1. 0.124 | | |

S19b Table. *The effects of consensus messaging on COVID-19 vaccination intention in Study 3 (subjects with low consensus perception and speeders are included; we do not control for demographic variables).*

|  | Pre-post: Vax. int. | | | Post-only: Vax. int. | | | Pre-post: Boost. int. | | | Post-only: Boost. int. | | |
| --- | --- | --- | --- | --- | --- | --- | --- | --- | --- | --- | --- | --- |
|  | *b* | *SE* | *p* | *b* | *SE* | *p* | *b* | *SE* | *p* | *b* | *SE* | *p* |
| Intercept | -0.02 | 0.03 | 0.420 | 1.57 | 0.10 | <0.001 | 0.00 | 0.04 | 1.000 | 3.56 | 0.09 | <0.001 |
| Message [Consensus] | 0.05 | 0.04 | 0.251 | 0.17 | 0.14 | 0.228 | -0.07 | 0.06 | 0.239 | 0.18 | 0.14 | 0.184 |
| Observations | 167 | | | 175 | | | 355 | | | 376 | | |
| R^2^ / R^2^ adjusted | 0.008 / 0.002 | | | 0.008 / 0.003 | | | 0.004 / 0.001 | | | 0.005 / 0.002 | | |

### S3.2.3. Moderators of the effects of consensus messaging on vaccine attitudes

We examined whether factors such as ideology, trust in scientists, and priors moderate the effects of consensus messaging on vaccine attitudes. In the “pre-post” design condition, all the effects of ideology were non-significant, whereas in the “post-only” design condition, right-wing ideology was related to higher worry, more negative belief, and lower policy support. Importantly, the interactive effects between consensus messaging and ideology were all non-significant. Similarly, the main and moderating effects of trust in science were non-significant across all DVs in the “pre-post” design condition, but exerted only main effects in the “post-only” design condition. Specifically, trust in scientists was associated with lower worry, more positive belief, and higher policy support. Finally, prior attitudes turned out to be a negative predictor of change in worry and belief (the higher the priors, the smaller the change in the attitudes) and non-significant for policy support. The details of these analyses are shown in S20a-b (ideology), S21a-b (trust), and S22a-b (priors) Tables.

S20a Table. *The effects of consensus messaging and ideology on vaccine worry, belief, and policy support in Study 3*

|  | Pre-post: Worry | | | Post-only: Worry | | | Pre-post: Belief | | | Post-only: Belief | | | Pre-post: Policy support | | | Post-only: Policy support | | |
| --- | --- | --- | --- | --- | --- | --- | --- | --- | --- | --- | --- | --- | --- | --- | --- | --- | --- | --- |
|  | *b* | *SE* | *p* | *b* | *SE* | *p* | *b* | *SE* | *p* | *b* | *SE* | *p* | *b* | *SE* | *p* | *b* | *SE* | *p* |
| Intercept | -0.04 | 0.07 | 0.579 | 3.45 | 0.16 | <0.001 | -0.06 | 0.09 | 0.495 | 2.61 | 0.15 | <0.001 | -0.06 | 0.05 | 0.162 | 1.91 | 0.18 | <0.001 |
| Age | 0.11 | 0.11 | 0.319 | -1.55 | 0.24 | <0.001 | 0.05 | 0.13 | 0.695 | 1.73 | 0.23 | <0.001 | 0.10 | 0.07 | 0.146 | 2.49 | 0.28 | <0.001 |
| Gender | -0.07 | 0.04 | 0.142 | 0.36 | 0.10 | <0.001 | 0.07 | 0.05 | 0.175 | -0.22 | 0.09 | 0.022 | -0.02 | 0.03 | 0.476 | -0.33 | 0.11 | 0.003 |
| Education | 0.06 | 0.08 | 0.459 | -0.53 | 0.16 | 0.001 | 0.03 | 0.09 | 0.754 | 0.42 | 0.16 | 0.008 | 0.02 | 0.05 | 0.652 | 0.56 | 0.19 | 0.003 |
| Message [Consensus] | -0.01 | 0.04 | 0.817 | 0.04 | 0.10 | 0.704 | -0.00 | 0.05 | 0.982 | -0.05 | 0.09 | 0.550 | -0.01 | 0.03 | 0.824 | -0.06 | 0.11 | 0.581 |
| Ideology | -0.10 | 0.14 | 0.461 | 1.55 | 0.32 | <0.001 | 0.08 | 0.17 | 0.616 | -0.84 | 0.30 | 0.006 | -0.08 | 0.09 | 0.363 | -0.97 | 0.36 | 0.007 |
| Message x Ideology | -0.21 | 0.20 | 0.298 | -0.02 | 0.43 | 0.967 | 0.12 | 0.24 | 0.616 | -0.67 | 0.42 | 0.105 | 0.07 | 0.13 | 0.604 | -0.19 | 0.49 | 0.700 |
| Observations | 508 | | | 543 | | | 508 | | | 543 | | | 508 | | | 543 | | |
| R^2^ / R^2^ adjusted | 0.018 / 0.006 | | | 0.186 / 0.177 | | | 0.008 / -0.004 | | | 0.171 / 0.162 | | | 0.008 / -0.004 | | | 0.188 / 0.179 | | |

S20b Table. *The effects of consensus messaging and ideology on vaccine worry, belief, and policy support in Study 3 (subjects with low consensus perception and speeders are included; we do not control for demographic variables).*

|  | Pre-post: Worry | | | Post-only: Worry | | | Pre-post: Beliefs | | | Post-only: Beliefs | | | Pre-post: Policy support | | | Post-only: Policy support | | |
| --- | --- | --- | --- | --- | --- | --- | --- | --- | --- | --- | --- | --- | --- | --- | --- | --- | --- | --- |
|  | *b* | *SE* | *p* | *b* | *SE* | *p* | *b* | *SE* | *p* | *b* | *SE* | *p* | *b* | *SE* | *p* | *b* | *SE* | *p* |
| Intercept | -0.02 | 0.03 | 0.624 | 2.81 | 0.07 | <0.001 | 0.02 | 0.04 | 0.666 | 3.33 | 0.07 | <0.001 | -0.03 | 0.02 | 0.147 | 2.92 | 0.08 | <0.001 |
| Message [Consensus] | 0.01 | 0.04 | 0.859 | 0.01 | 0.10 | 0.950 | -0.00 | 0.05 | 0.958 | -0.04 | 0.10 | 0.712 | -0.01 | 0.03 | 0.835 | -0.01 | 0.12 | 0.907 |
| Ideology | -0.22 | 0.14 | 0.119 | 1.83 | 0.32 | <0.001 | 0.10 | 0.16 | 0.547 | -1.14 | 0.31 | <0.001 | -0.09 | 0.09 | 0.281 | -1.38 | 0.38 | <0.001 |
| Message x Ideology | -0.08 | 0.20 | 0.688 | -0.12 | 0.45 | 0.785 | 0.09 | 0.23 | 0.706 | -0.60 | 0.44 | 0.166 | 0.07 | 0.12 | 0.596 | -0.01 | 0.53 | 0.985 |
| Observations | 522 | | | 551 | | | 522 | | | 551 | | | 522 | | | 551 | | |
| R^2^ / R^2^ adjusted | 0.013 / 0.007 | | | 0.102 / 0.097 | | | 0.003 / -0.003 | | | 0.079 / 0.074 | | | 0.002 / -0.003 | | | 0.049 / 0.044 | | |

S21a Table. *The effects of consensus messaging and trust in scientists on vaccine worry, belief, and policy support in Study 3.*

|  | Pre-post: Worry | | | Post-only: Worry | | | Pre-post: Belief | | | Post-only: Belief | | | Pre-post: Policy support | | | Post-only: Policy support | | |
| --- | --- | --- | --- | --- | --- | --- | --- | --- | --- | --- | --- | --- | --- | --- | --- | --- | --- | --- |
|  | *b* | *SE* | *p* | *b* | *SE* | *p* | *b* | *SE* | *p* | *b* | *SE* | *p* | *b* | *SE* | *P* | *b* | *SE* | *p* |
| Intercept | -0.04 | 0.07 | 0.539 | 3.27 | 0.12 | <0.001 | -0.05 | 0.09 | 0.542 | 2.85 | 0.11 | <0.001 | -0.07 | 0.05 | 0.143 | 2.22 | 0.13 | <0.001 |
| Age | 0.09 | 0.12 | 0.417 | -1.02 | 0.19 | <0.001 | 0.06 | 0.14 | 0.654 | 1.13 | 0.17 | <0.001 | 0.11 | 0.07 | 0.146 | 1.79 | 0.21 | <0.001 |
| Gender | -0.06 | 0.04 | 0.159 | 0.12 | 0.08 | 0.118 | 0.07 | 0.05 | 0.182 | 0.01 | 0.07 | 0.866 | -0.02 | 0.03 | 0.474 | -0.08 | 0.08 | 0.340 |
| Education | 0.07 | 0.08 | 0.335 | -0.31 | 0.13 | 0.016 | 0.01 | 0.09 | 0.879 | 0.14 | 0.11 | 0.216 | 0.03 | 0.05 | 0.586 | 0.21 | 0.14 | 0.138 |
| Message [Consensus] | -0.01 | 0.04 | 0.895 | -0.01 | 0.07 | 0.900 | -0.00 | 0.05 | 0.963 | -0.01 | 0.07 | 0.934 | -0.01 | 0.03 | 0.809 | 0.00 | 0.08 | 0.972 |
| Trust in scientists | 0.14 | 0.12 | 0.241 | -3.00 | 0.20 | <0.001 | 0.01 | 0.14 | 0.930 | 3.04 | 0.18 | <0.001 | -0.02 | 0.08 | 0.840 | 3.48 | 0.22 | <0.001 |
| Message x Trust | -0.05 | 0.16 | 0.762 | 0.25 | 0.27 | 0.356 | -0.14 | 0.19 | 0.463 | -0.25 | 0.24 | 0.309 | 0.06 | 0.10 | 0.572 | -0.53 | 0.30 | 0.074 |
| Observations | 508 | | | 543 | | | 508 | | | 543 | | | 508 | | | 543 | | |
| R^2^ / R^2^ adjusted | 0.012 / -0.000 | | | 0.502 / 0.496 | | | 0.006 / -0.006 | | | 0.559 / 0.554 | | | 0.007 / -0.005 | | | 0.540 / 0.535 | | |

S21b Table. *The effects of consensus messaging and trust in scientists on vaccine worry, belief, and policy support in Study 3 (subjects with low consensus perception and speeders are included; we do not control for demographic variables).*

|  | Pre-post: Worry | | | Post-only: Worry | | | Pre-post: Beliefs | | | Post-only: Beliefs | | | Pre-post: Policy support | | | Post-only: Policy support | | |
| --- | --- | --- | --- | --- | --- | --- | --- | --- | --- | --- | --- | --- | --- | --- | --- | --- | --- | --- |
|  | *b* | *SE* | *p* | *b* | *SE* | *p* | *b* | *SE* | *p* | *b* | *SE* | *p* | *b* | *SE* | *p* | *b* | *SE* | *p* |
| Intercept | -0.01 | 0.03 | 0.635 | 2.82 | 0.05 | <0.001 | 0.02 | 0.04 | 0.663 | 3.32 | 0.05 | <0.001 | -0.03 | 0.02 | 0.144 | 2.90 | 0.06 | <0.001 |
| Message [Consensus] | 0.01 | 0.04 | 0.804 | -0.03 | 0.08 | 0.708 | -0.00 | 0.05 | 0.937 | -0.00 | 0.07 | 0.997 | -0.01 | 0.03 | 0.822 | 0.04 | 0.09 | 0.676 |
| Trust | 0.26 | 0.12 | 0.024 | -3.27 | 0.20 | <0.001 | -0.00 | 0.13 | 0.973 | 3.19 | 0.18 | <0.001 | -0.02 | 0.07 | 0.765 | 3.84 | 0.22 | <0.001 |
| Message x Trust | -0.16 | 0.16 | 0.335 | 0.43 | 0.28 | 0.123 | -0.11 | 0.19 | 0.552 | -0.33 | 0.26 | 0.193 | 0.08 | 0.10 | 0.411 | -0.71 | 0.31 | 0.023 |
| Observations | 522 | | | 551 | | | 522 | | | 551 | | | 522 | | | 551 | | |
| R^2^ / R^2^ adjusted | 0.012 / 0.006 | | | 0.475 / 0.472 | | | 0.002 / -0.004 | | | 0.507 / 0.505 | | | 0.002 / -0.004 | | | 0.480 / 0.477 | | |

S22a Table. *The effects of consensus messaging and priors on vaccine worry, belief, and policy support in “pre-post” design condition in Study 3*

|  | Pre-post: Worry | | | Pre-post: Belief | | | Pre-post: Policy support | | |
| --- | --- | --- | --- | --- | --- | --- | --- | --- | --- |
|  | *b* | *SE* | *p* | *b* | *SE* | *p* | *b* | *SE* | *p* |
| Intercept | 0.03 | 0.07 | 0.646 | -0.15 | 0.08 | 0.069 | -0.06 | 0.05 | 0.176 |
| Age | -0.06 | 0.12 | 0.600 | 0.26 | 0.14 | 0.058 | 0.10 | 0.08 | 0.212 |
| Gender | -0.05 | 0.04 | 0.217 | 0.06 | 0.05 | 0.229 | -0.02 | 0.03 | 0.483 |
| Education | 0.03 | 0.07 | 0.706 | 0.07 | 0.09 | 0.393 | 0.03 | 0.05 | 0.593 |
| Message [Consensus] | -0.01 | 0.04 | 0.764 | 0.01 | 0.05 | 0.904 | -0.01 | 0.03 | 0.826 |
| Priors | -0.30 | 0.09 | 0.001 | -0.37 | 0.12 | 0.002 | 0.05 | 0.06 | 0.362 |
| Message x Priors | -0.17 | 0.13 | 0.183 | -0.18 | 0.17 | 0.288 | -0.05 | 0.08 | 0.556 |
| Observations | 508 | | | 508 | | | 508 | | |
| R^2^ / R^2^ adjusted | 0.070 / 0.059 | | | 0.057 / 0.046 | | | 0.008 / -0.004 | | |

*Note. In each analysis, priors were the responses to the respective DV prior to manipulation, e.g., prior ratings of worry in the analysis of worry change scores.*

S22b Table. *The effects of consensus messaging and priors on vaccine worry, belief, and policy support in “pre-post” design condition in Study 3 (subjects with low consensus perception and speeders are included; we do not control for demographic variables).*

|  | Pre-post: Worry | | | Pre-post: Beliefs | | | Pre-post: Policy support | | |
| --- | --- | --- | --- | --- | --- | --- | --- | --- | --- |
|  | *b* | *SE* | *p* | *b* | *SE* | *p* | *b* | *SE* | *p* |
| Intercept | -0.01 | 0.03 | 0.711 | 0.01 | 0.04 | 0.735 | -0.03 | 0.02 | 0.149 |
| Message [Consensus] | 0.00 | 0.04 | 0.928 | 0.00 | 0.05 | 0.959 | -0.01 | 0.03 | 0.844 |
| Priors | -0.34 | 0.09 | <0.001 | -0.32 | 0.11 | 0.005 | 0.06 | 0.05 | 0.299 |
| Message x Priors | -0.10 | 0.13 | 0.430 | -0.13 | 0.16 | 0.431 | -0.03 | 0.08 | 0.685 |
| Observations | 522 | | | 522 | | | 522 | | |
| R^2^ / R^2^ adjusted | 0.068 / 0.062 | | | 0.042 / 0.037 | | | 0.003 / -0.003 | | |

*Note. In each analysis, priors were the responses to the respective DV prior to manipulation, e.g., prior ratings of worry in the analysis of worry change scores.*

### S3.2.4 Moderators of the effects of consensus messaging on COVID-19 vaccination intentions

We investigated the moderating role of ideology, trust in scientists, and priors in the relationship between consensus messaging and COVID-19 vaccination intentions. Ideology effects were non-significant for intentions to receive a vaccine both in the “pre-post” and “post-only” design conditions. For the intentions to receive a booster shot, we found the main as well as interactive effects of ideology in the “pre-post” design condition. Rather surprisingly, right-wing ideology was related to larger changes in intentions to receive a booster shot; this main effect was qualified by an interaction with consensus messaging. As shown in S4 Fig, in the control condition, those on the political Left changed their intention to receive a booster shot less than the political Right (b = -0.18, SE = 0.08, t(341) = -2.215, p = .027), whereas for the consensus messaging condition, the differences between the political Left and Right were non-significant (b = 0.07, SE = 0.08, t(341) = 0.79, p = 0.428). However, all the confidence intervals for the +/-1 SD of ideology included zero, suggesting that the change was not significantly different from zero. Next, we analyzed the effects of ideology in the “post-only” design condition and found only its main effects: right-wing political ideology stances were related to lower intentions to receive a booster shot. The model details are presented in S23a Table.

With regard to trust in scientists, we only found a (main) positive effect of trust in scientists in the “post-only” design condition for both intentions to receive a vaccine and a booster shot. Any effects of trust in scientists were non-significant in the “pre-post” design condition. The details are in S24a Table. For the analysis of the moderating effects of priors, we found the main effects for both intentions to receive a first or a booster shot of vaccine: higher priors were related to lower changes in intentions. Furthermore, for the intentions to vaccinate, we found interaction between priors and consensus messaging: as shown in S5 Fig, in the control condition, change in the intentions of people with higher priors was negative (suggesting decreased intentions) and also lower compared to people with low priors (b = 0.21, SE = 0.06, t(153) = 3.42, p = .001), but in the consensus messaging, the difference was non-significant (b = 0.00, SE = (0.06), t(153) = 0.02, p = .986). The details are in S25a Table.


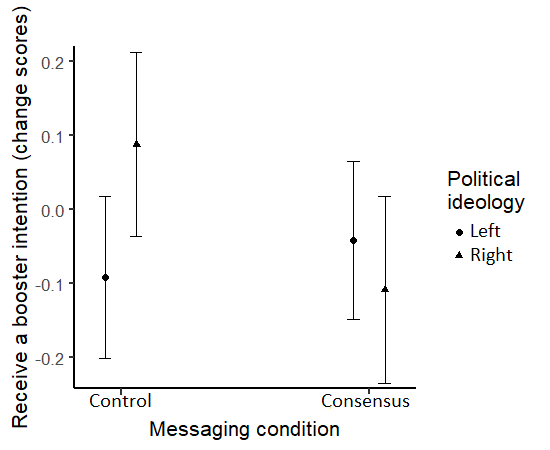


S4 Fig. The effects of consensus messaging and political ideology on the intention to receive a booster shot in Study 3 (in “pre-post” design condition).


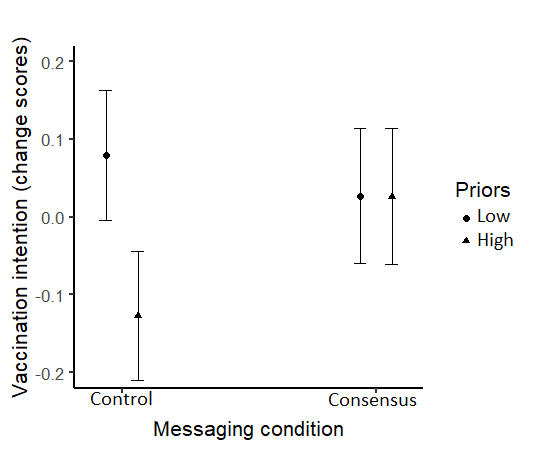


S5 Fig. The effects of consensus messaging and prior vaccination intention among the unvaccinated in Study 3 (in the “pre-post” design condition).

S23a Table. *The effects of consensus messaging and ideology on COVID-19 vaccination intentions in Study 3*

|  | Pre-post: Vax. int. | | | Post-only: Vax. int. | | | Pre-post: Boost. int. | | | Post-only: Boost. int. | | |
| --- | --- | --- | --- | --- | --- | --- | --- | --- | --- | --- | --- | --- |
|  | *b* | *SE* | *p* | *b* | *SE* | *p* | *b* | *SE* | *p* | *b* | *SE* | *p* |
| Intercept | -0.03 | 0.07 | 0.653 | 1.51 | 0.23 | <0.001 | -0.06 | 0.11 | 0.591 | 2.47 | 0.22 | <0.001 |
| Age | -0.06 | 0.12 | 0.602 | 0.32 | 0.39 | 0.416 | 0.23 | 0.16 | 0.145 | 2.19 | 0.33 | <0.001 |
| Gender | -0.01 | 0.05 | 0.861 | -0.07 | 0.14 | 0.638 | -0.01 | 0.06 | 0.885 | -0.20 | 0.13 | 0.121 |
| Education | 0.03 | 0.08 | 0.718 | 0.03 | 0.26 | 0.897 | -0.04 | 0.10 | 0.723 | 0.56 | 0.22 | 0.009 |
| Message [Consensus] | 0.06 | 0.05 | 0.184 | 0.03 | 0.15 | 0.834 | -0.08 | 0.06 | 0.197 | 0.16 | 0.13 | 0.211 |
| Ideology | 0.15 | 0.19 | 0.437 | 0.32 | 0.58 | 0.577 | 0.41 | 0.18 | 0.027 | -1.03 | 0.40 | 0.010 |
| Message x Ideology | -0.13 | 0.25 | 0.618 | 0.42 | 0.75 | 0.574 | -0.56 | 0.26 | 0.034 | 0.70 | 0.57 | 0.220 |
| Observations | 160 | | | 168 | | | 348 | | | 375 | | |
| R^2^ / R^2^ adjusted | 0.017 / -0.022 | | | 0.027 / -0.009 | | | 0.026 / 0.009 | | | 0.150 / 0.137 | | |

S23b Table. *The effects of consensus messaging and ideology on COVID-19 vaccination intentions in Study 3 (subjects with low consensus perception and speeders are included; we do not control for demographic variables).*

|  | Pre-post: Vax. int. | | | Post-only: Vax. int. | | | Pre-post: Boost. int. | | | Post-only: Boost. int. | | |
| --- | --- | --- | --- | --- | --- | --- | --- | --- | --- | --- | --- | --- |
|  | *b* | *SE* | *p* | *b* | *SE* | *p* | *b* | *SE* | *p* | *b* | *SE* | *p* |
| Intercept | -0.04 | 0.03 | 0.273 | 1.55 | 0.11 | <0.001 | 0.02 | 0.04 | 0.702 | 3.53 | 0.09 | <0.001 |
| Message [Consensus] | 0.06 | 0.04 | 0.183 | 0.08 | 0.15 | 0.583 | -0.09 | 0.06 | 0.125 | 0.21 | 0.14 | 0.130 |
| Ideology | 0.15 | 0.18 | 0.398 | 0.26 | 0.56 | 0.647 | 0.43 | 0.18 | 0.017 | -1.42 | 0.41 | 0.001 |
| Message x Ideology | -0.14 | 0.24 | 0.558 | 0.63 | 0.74 | 0.394 | -0.56 | 0.26 | 0.034 | 0.95 | 0.60 | 0.114 |
| Observations | 167 | | | 175 | | | 355 | | | 376 | | |
| R^2^ / R^2^ adjusted | 0.012 / -0.006 | | | 0.029 / 0.012 | | | 0.021 / 0.013 | | | 0.038 / 0.030 | | |

S24a Table. *The effects of consensus messaging and trust in scientists on COVID-19 vaccination intentions in Study 3*

|  | Pre-post: Vax. int. | | | Post-only: Vax. int. | | | Pre-post: Boost. int. | | | Post-only: Boost. int. | | |
| --- | --- | --- | --- | --- | --- | --- | --- | --- | --- | --- | --- | --- |
|  | *b* | *SE* | *p* | *b* | *SE* | *p* | *b* | *SE* | *p* | *b* | *SE* | *p* |
| Intercept | 0.00 | 0.07 | 0.972 | 1.79 | 0.21 | <0.001 | -0.04 | 0.11 | 0.704 | 2.48 | 0.19 | <0.001 |
| Age | -0.08 | 0.12 | 0.497 | 0.33 | 0.36 | 0.348 | 0.20 | 0.16 | 0.222 | 1.93 | 0.29 | <0.001 |
| Gender | 0.01 | 0.05 | 0.781 | -0.05 | 0.13 | 0.696 | -0.00 | 0.06 | 0.960 | 0.01 | 0.12 | 0.905 |
| Education | 0.06 | 0.08 | 0.486 | 0.20 | 0.24 | 0.408 | -0.05 | 0.10 | 0.623 | 0.26 | 0.19 | 0.178 |
| Message [Consensus] | 0.03 | 0.05 | 0.559 | -0.00 | 0.16 | 0.978 | -0.09 | 0.06 | 0.151 | 0.17 | 0.12 | 0.141 |
| Trust in scientists | 0.26 | 0.13 | 0.052 | 2.05 | 0.40 | <0.001 | -0.18 | 0.18 | 0.330 | 2.56 | 0.32 | <0.001 |
| Message x Trust | -0.11 | 0.19 | 0.567 | -0.80 | 0.54 | 0.137 | 0.40 | 0.25 | 0.104 | -0.12 | 0.44 | 0.784 |
| Observations | 160 | | | 168 | | | 348 | | | 375 | | |
| R^2^ / R^2^ adjusted | 0.042 / 0.005 | | | 0.197 / 0.167 | | | 0.018 / 0.001 | | | 0.348 / 0.338 | | |

S24b Table. *The effects of consensus messaging and trust in scientists on COVID-19 vaccination intentions in Study 3 (subjects with low consensus perception and speeders are included; we do not control for demographic variables).*

|  | Pre-post: Vax. int. | | | Post-only: Vax. int. | | | Pre-post: Boost. int. | | | Post-only: Boost. int. | | |
| --- | --- | --- | --- | --- | --- | --- | --- | --- | --- | --- | --- | --- |
|  | *b* | *SE* | *p* | *b* | *SE* | *p* | *b* | *SE* | *p* | *b* | *SE* | *p* |
| Intercept | 0.01 | 0.04 | 0.707 | 1.92 | 0.11 | <0.001 | 0.01 | 0.05 | 0.796 | 3.33 | 0.09 | <0.001 |
| Message [Consensus] | 0.03 | 0.05 | 0.581 | 0.10 | 0.16 | 0.534 | -0.11 | 0.06 | 0.096 | 0.24 | 0.12 | 0.048 |
| Trust | 0.22 | 0.12 | 0.077 | 1.98 | 0.39 | <0.001 | -0.15 | 0.18 | 0.405 | 2.92 | 0.33 | <0.001 |
| Message x Trust | -0.11 | 0.18 | 0.537 | -0.50 | 0.52 | 0.342 | 0.38 | 0.24 | 0.114 | -0.47 | 0.47 | 0.316 |
| Observations | 167 | | | 175 | | | 355 | | | 376 | | |
| R^2^ / R^2^ adjusted | 0.031 / 0.013 | | | 0.210 / 0.197 | | | 0.012 / 0.003 | | | 0.267 / 0.262 | | |

S25a Table. *The effects of consensus messaging and priors on COVID-19 vaccination intentions in “pre-post” design condition in Study 3*

|  | Pre-post: Vax. int. | | | Pre-post: Boost. int. | | |
| --- | --- | --- | --- | --- | --- | --- |
|  | *b* | *SE* | *p* | *b* | *SE* | *p* |
| Intercept | -0.00 | 0.07 | 0.975 | -0.19 | 0.11 | 0.078 |
| Age | -0.09 | 0.12 | 0.443 | 0.50 | 0.17 | 0.003 |
| Gender | -0.00 | 0.04 | 0.946 | -0.04 | 0.06 | 0.544 |
| Education | 0.01 | 0.08 | 0.907 | 0.00 | 0.10 | 0.997 |
| Message [Consensus] | 0.05 | 0.04 | 0.240 | -0.04 | 0.06 | 0.469 |
| Priors | -0.49 | 0.14 | 0.001 | -0.53 | 0.13 | <0.001 |
| Message x Priors | 0.49 | 0.21 | 0.020 | 0.16 | 0.17 | 0.364 |
| Observations | 160 | | | 348 | | |
| R^2^ / R^2^ adjusted | 0.083 / 0.047 | | | - 1. 0.060 | | |

*Note. In each analysis, priors were the responses to the respective DV prior to manipulation, e.g., prior ratings of worry in the analysis of worry change scores.*

S25b Table. *The effects of consensus messaging and priors on COVID-19 vaccination intentions in “pre-post” design condition in Study 3 (subjects with low consensus perception and speeders are included; we do not control for demographic variables).*

|  | Pre-post: Vax. int. | | | Pre-post: Boost. int. | | |
| --- | --- | --- | --- | --- | --- | --- |
|  | *b* | *SE* | *p* | *b* | *SE* | *p* |
| Intercept | -0.02 | 0.03 | 0.442 | -0.01 | 0.04 | 0.870 |
| Message [Consensus] | 0.05 | 0.04 | 0.254 | -0.06 | 0.06 | 0.303 |
| Priors | -0.48 | 0.14 | 0.001 | -0.46 | 0.12 | <0.001 |
| Message x Priors | 0.48 | 0.20 | 0.017 | 0.22 | 0.17 | 0.192 |
| Observations | 167 | | | 355 | | |
| R^2^ / R^2^ adjusted | 0.078 / 0.061 | | | 0.052 / 0.044 | | |

*Note. In each analysis, priors were the responses to the respective DV prior to manipulation, e.g., prior ratings of worry in the analysis of worry change scores.*

## S3.3. Testing hypotheses with SEM

S26 Table. *Fit of SEM models in Study 3.*

|  | Pre-post design | | Post-only design | |
| --- | --- | --- | --- | --- |
| Final variable in the model🡪 | Policy support | Vaccination intention | Policy support | Vaccination intention |
| χ^2^(4) | 9.52 | 2.73 | 13.25 | 21.45 |
| p | .049 | .605 | .010 | .000 |
| CFI | 0.89 | 1.00 | 0.99 | 0.99 |
| SRMR | 0.03 | 0.02 | 0.05 | 0.05 |
| RMSEA | 0.05 | 0.00 | 0.07 | 0.09 |
| 95%CI RMSEA | (0.00 – 0.10) | (0.00 – 0.06) | (0.03 – 0.11) | (0.06 – 0.13) |

*Note. In the models with vaccination intention as the final variable, we collapsed data across intention to vaccinate with the first dose and intention to receive a booster shot in the future.*

### **S3.3.1. Pre-post design condition**

Here we analyze the responses from the pre-post design condition, i.e., the change scores between pre- and post-messaging responses. None of the paths (direct or indirect) were found to be statistically significant.

S27 Table. *Path coefficients and indirect effects for policy support as final variable in the model for the pre-post design condition in Study 3*

|  |  |  |  |  |  |  |  | 95% CI | |
| --- | --- | --- | --- | --- | --- | --- | --- | --- | --- |
| Paths |  |  |  | *b* | SE | *z* | *p* | Lower | Upper |
| Consensus messaging | → | Perceived scientific consensus |  | 0.06 | 0.01 | 6.12 | .000 | 0.04 | 0.08 |
| Perceived scientific consensus | → | Belief |  | 0.00 | 0.04 | -0.11 | .912 | -0.07 | 0.07 |
| Perceived scientific consensus | → | Worry |  | -0.04 | 0.06 | -0.64 | .524 | -0.15 | 0.08 |
| Belief | → | Worry |  | -0.16 | 0.08 | -1.98 | .047 | -0.32 | 0.00 |
| Belief | → | Policy support |  | 0.10 | 0.04 | 2.34 | .019 | 0.03 | 0.20 |
| Worry | → | Policy support |  | -0.02 | 0.05 | -0.33 | .740 | -0.12 | 0.09 |
| *Indirect effects of perceived consensus* |  |  |  |  |  |  |  |  |  |
| Through belief |  |  |  | 0.00 | 0.00 | -0.10 | .918 | -0.01 | 0.01 |
| Through worry |  |  |  | 0.00 | 0.00 | 0.17 | .862 | 0.00 | 0.01 |
| Through belief and worry |  |  |  | 0.00 | 0.00 | -0.03 | .975 | 0.00 | 0.00 |
| Combined indirect effect |  |  |  | 0.00 | 0.01 | 0.04 | .968 | -0.01 | 0.01 |
| *Indirect effects of consensus messaging* |  |  |  |  |  |  |  |  |  |
| Through perceived consensus and belief |  |  |  | 0.00 | 0.00 | -0.10 | .918 | 0.00 | 0.00 |
| Through perceived consensus and worry |  |  |  | 0.00 | 0.00 | 0.17 | .865 | 0.00 | 0.00 |
| Through perceived consensus, belief, and worry |  |  |  | 0.00 | 0.00 | -0.03 | .975 | 0.00 | 0.00 |
| Combined indirect effect |  |  |  | 0.00 | 0.00 | 0.04 | .968 | 0.00 | 0.00 |

S28 Table. *Path coefficients and indirect effects for vaccination intention as final variable in the model for the pre-post design condition in Study 3*

|  |  |  |  |  |  |  |  | 95% CI | |
| --- | --- | --- | --- | --- | --- | --- | --- | --- | --- |
| Paths |  |  |  | *b* | SE | *z* | *p* | Lower | Upper |
| Consensus messaging | → | Perceived scientific consensus |  | 0.06 | 0.01 | 6.11 | .000 | 0.04 | 0.08 |
| Perceived scientific consensus | → | Belief |  | 0.00 | 0.04 | -0.11 | .912 | -0.08 | 0.07 |
| Perceived scientific consensus | → | Worry |  | -0.04 | 0.06 | -0.62 | .532 | -0.16 | 0.07 |
| Belief | → | Worry |  | -0.16 | 0.08 | -2.03 | .043 | -0.31 | -0.01 |
| Belief | → | Policy support |  | 0.04 | 0.03 | 1.45 | .147 | 0.00 | 0.12 |
| Worry | → | Policy support |  | -0.02 | 0.02 | -1.26 | .207 | -0.07 | 0.01 |
| *Indirect effects of perceived consensus* |  |  |  |  |  |  |  |  |  |
| Through belief |  |  |  | 0.00 | 0.00 | -0.09 | .931 | -0.01 | 0.00 |
| Through worry |  |  |  | 0.00 | 0.00 | 0.47 | .640 | 0.00 | 0.01 |
| Through belief and worry |  |  |  | 0.00 | 0.00 | -0.08 | .937 | 0.00 | 0.00 |
| Combined indirect effect |  |  |  | 0.00 | 0.00 | 0.24 | .812 | 0.00 | 0.01 |
| *Indirect effects of consensus messaging* |  |  |  |  |  |  |  |  |  |
| Through perceived consensus and belief |  |  |  | 0.00 | 0.00 | -0.09 | .931 | 0.00 | 0.00 |
| Through perceived consensus and worry |  |  |  | 0.00 | 0.00 | 0.46 | .648 | 0.00 | 0.00 |
| Through perceived consensus, belief, and worry |  |  |  | 0.00 | 0.00 | -0.08 | .936 | 0.00 | 0.00 |
| Combined indirect effect |  |  |  | 0.00 | 0.00 | 0.24 | .813 | 0.00 | 0.00 |

### **S3.3.2. Post-only design condition**

Here we analyze the responses from the post-only design condition, i.e., the responses were given just once after consensus/control messaging. In other words, we do not analyze change scores here.

S29 Table. *Path coefficients and indirect effects for* ***policy support*** *as final variable in the model for* ***the post-only*** *design condition in Study 3*

|  |  |  |  |  |  |  |  | 95% CI | |
| --- | --- | --- | --- | --- | --- | --- | --- | --- | --- |
| Paths |  |  |  | *b* | SE | *z* | *p* | Lower | Upper |
| Consensus messaging | → | Perceived scientific consensus |  | 0.10 | 0.02 | 5.26 | <.001 | 0.06 | 0.14 |
| Perceived scientific consensus | → | Belief |  | 0.53 | 0.05 | 10.67 | <.001 | 0.43 | 0.63 |
| Perceived scientific consensus | → | Worry |  | -0.16 | 0.04 | -3.88 | <.001 | -0.23 | -0.08 |
| Belief | → | Worry |  | -0.79 | 0.03 | -28.18 | <.001 | -0.84 | -0.73 |
| Belief | → | Policy support |  | 0.67 | 0.05 | 12.51 | <.001 | 0.56 | 0.77 |
| Worry | → | Policy support |  | -0.38 | 0.05 | -7.19 | <.001 | -0.48 | -0.27 |
| ***Indirect effects of perceived consensus*** |  |  |  |  |  |  |  |  |  |
| Through belief |  |  |  | 0.36 | 0.04 | 7.96 | <.001 | 0.28 | 0.45 |
| Through worry |  |  |  | 0.06 | 0.02 | 3.31 | 0.001 | 0.03 | 0.10 |
| Through belief and worry |  |  |  | 0.16 | 0.03 | 5.80 | <.001 | 0.11 | 0.22 |
| Combined indirect effect |  |  |  | 0.57 | 0.05 | 11.57 | <.001 | 0.47 | 0.67 |
| ***Indirect effects of consensus messaging*** |  |  |  |  |  |  |  |  |  |
| Through perceived consensus and belief |  |  |  | 0.04 | 0.01 | 4.59 | <.001 | 0.02 | 0.05 |
| Through perceived consensus and worry |  |  |  | 0.01 | 0.00 | 2.72 | .007 | 0.00 | 0.01 |
| Through perceived consensus, belief, and worry |  |  |  | 0.02 | 0.00 | 3.82 | <.001 | 0.01 | 0.03 |
| Combined indirect effect |  |  |  | 0.06 | 0.01 | 4.88 | <.001 | 0.04 | 0.08 |

S30 Table. *Path coefficients and indirect effects for* ***vaccination intention*** *as final variable in the model for the* ***post-only design*** *condition in Study 3*

|  |  |  |  |  |  |  |  | 95% CI | |
| --- | --- | --- | --- | --- | --- | --- | --- | --- | --- |
| Paths |  |  | label | *b* | SE | *z* | *p* | Lower | Upper |
| Consensus messaging | → | Perceived scientific consensus |  | 0.10 | 0.02 | 5.10 | <.001 | 0.06 | 0.14 |
| Perceived scientific consensus | → | Belief |  | 0.53 | 0.05 | 10.66 | <.001 | 0.44 | 0.63 |
| Perceived scientific consensus | → | Worry |  | -0.16 | 0.04 | -3.95 | <.001 | -0.23 | -0.08 |
| Belief | → | Worry |  | -0.79 | 0.03 | -28.44 | <.001 | -0.84 | -0.73 |
| Belief | → | Policy support |  | 0.42 | 0.07 | 6.42 | <.001 | 0.29 | 0.55 |
| Worry | → | Policy support |  | -0.64 | 0.06 | -10.07 | <.001 | -0.76 | -0.51 |
| ***Indirect effects of perceived consensus*** |  |  |  |  |  |  |  |  |  |
| Through belief |  |  |  | 0.22 | 0.04 | 5.26 | <.001 | 0.15 | 0.32 |
| Through worry |  |  |  | 0.10 | 0.03 | 3.46 | .001 | 0.05 | 0.16 |
| Through belief and worry |  |  |  | 0.27 | 0.04 | 7.60 | <.001 | 0.20 | 0.34 |
| Combined indirect effect |  |  |  | 0.59 | 0.05 | 12.02 | <.001 | 0.50 | 0.69 |
| ***Indirect effects of consensus messaging*** |  |  |  |  |  |  |  |  |  |
| Through perceived consensus and belief |  |  |  | 0.02 | 0.01 | 3.76 | <.001 | 0.01 | 0.04 |
| Through perceived consensus and worry |  |  |  | 0.01 | 0.00 | 2.77 | .006 | 0.00 | 0.02 |
| Through perceived consensus, belief, and worry |  |  |  | 0.03 | 0.01 | 4.28 | <.001 | 0.02 | 0.04 |
| Combined indirect effect |  |  |  | 0.06 | 0.01 | 4.77 | <.001 | 0.04 | 0.08 |

## **S3.4. Analysis of psychological reactance**

### **S3.4.1. The effects of consensus messaging and moderator on psychological reactance**

S31a Table. *The effects of consensus messaging and* ***moderators*** *on* ***psychological reactance*** *in Study 3.*

|  | **Manipulations only** | | | **Ideology added** | | | **Trust added** | | | **Priors added** | | |
| --- | --- | --- | --- | --- | --- | --- | --- | --- | --- | --- | --- | --- |
|  | *b* | *SE* | *p* | *b* | *SE* | *p* | *b* | *SE* | *p* | *b* | *SE* | *p* |
| Intercept | 2.67 | 0.09 | **<0.001** | 2.53 | 0.09 | **<0.001** | 2.52 | 0.09 | **<0.001** | 2.54 | 0.12 | **<0.001** |
| Age | -0.46 | 0.14 | **0.001** | -0.31 | 0.14 | **0.020** | -0.12 | 0.13 | 0.377 | -0.10 | 0.19 | 0.596 |
| Gender | -0.03 | 0.06 | 0.635 | -0.02 | 0.05 | 0.722 | -0.09 | 0.05 | 0.082 | -0.03 | 0.07 | 0.693 |
| Education | -0.65 | 0.09 | **<0.001** | -0.47 | 0.09 | **<0.001** | -0.48 | 0.09 | **<0.001** | -0.57 | 0.12 | **<0.001** |
| Message [Consensus] | 0.42 | 0.05 | **<0.001** | 0.42 | 0.05 | **<0.001** | 0.42 | 0.05 | **<0.001** | 0.37 | 0.07 | **<0.001** |
| Design [Post-only] | 0.13 | 0.05 | **0.022** | 0.10 | 0.05 | 0.065 | 0.08 | 0.05 | 0.113 |  |  |  |
| Ideology |  |  |  | 0.69 | 0.21 | **0.001** |  |  |  |  |  |  |
| Ideology x Message |  |  |  | 0.48 | 0.24 | **0.044** |  |  |  |  |  |  |
| Ideology x Design |  |  |  | 0.47 | 0.24 | 0.050 |  |  |  |  |  |  |
| Trust in scientists |  |  |  |  |  |  | -0.73 | 0.14 | **<0.001** |  |  |  |
| Trust x Message |  |  |  |  |  |  | -1.03 | 0.19 | **<0.001** |  |  |  |
| Prior perceived consensus |  |  |  |  |  |  |  |  |  | -0.67 | 0.21 | **0.002** |
| Prior x Message |  |  |  |  |  |  |  |  |  | -1.25 | 0.29 | **<0.001** |
| Observations | 1051 | | | 1051 | | | 1051 | | | 508 | | |
| R^2^ / R^2^ adjusted | 0.102 / 0.098 | | | 0.182 / 0.175 | | | 0.253 / 0.248 | | | 0.240 / 0.231 | | |

For ideology, the 3-way interaction between both manipulations and political ideology was non-significant, thus we removed it and left only the 2-way interactions between conditions and ideology. As already mentioned in the main paper, ideology had a positive effect on reactance, i.e., the more ideologically right-wing the participant, the higher the reactance. The effect of ideology was however stronger in the consensus messaging condition (b = 1.41, SE = 0.17, 95%CI[1.08, 1.74]) as compared to the control condition (b = 0.92, SE = 0.17, 95%CI[0.59, 1.26]) and this difference was statistically significant (b = 0.48, SE = 0.24, t(1042) = 2.02, p = .044), see S6 Fig (Panel A). The effects of ideology on reactance were also somewhat stronger in the “post-only” (b = 1.40, SE = 0.17, 95%CI[1.07, 1.73]) as compared to “pre-post” design condition (b = 0.93, SE = 0.17, 95%CI[0.59, 1.27]); the difference was only marginally significant (b = 0.47, SE = 0.24, t(1042) = 1.96, p = 0.051), see S6 Fig (Panel B).

Turning to the effects of trust, we found negative effects of trust in scientists, i.e., the higher the trust, the lower the reactance. This effect was stronger in the consensus messaging condition (b = -1.77, SE = 0.13, 95%CI[-2.02, -1.51]) as compared to the control condition (b = -0.73, SE = 0.14, 95%CI[-1.00, -0.47] as shown in S7 Fig); this difference was statistically significant (b = 1.03, SE = 0.19, t(1043) = 5.57, <.001). Neither the main effect of design nor further interactions turned out to be statistically significant.

Finally, in the analysis of the moderating role of priors, we used (pre-messaging) perceived scientific consensus as priors. Using only observations from the “pre-post” design condition, we found that priors are negatively related to psychological reactance. As in the analysis of trust, these effects were stronger among people who received consensus messaging (b = -1.91, SE = 0.21, 95%CI[-2.32, -1.51]) compared to those who received control messaging (b = -0.67, SE = 0.21, 95%CI[-1.08, -0.25]), as shown in S8 Fig; this difference was statistically significant (b = 1.25, SE = 0.29, t(501) = 4.27, p < .001).


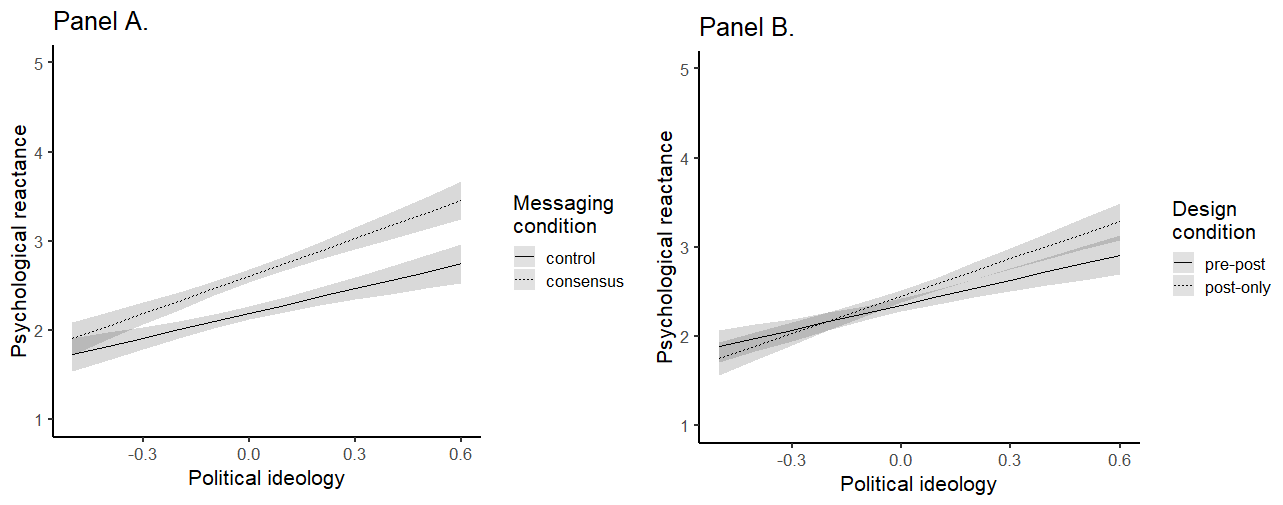


S6 Fig. The effects of political ideology and consensus messaging (Panel A) or design condition (Panel B) on psychological reactance in Study 3.


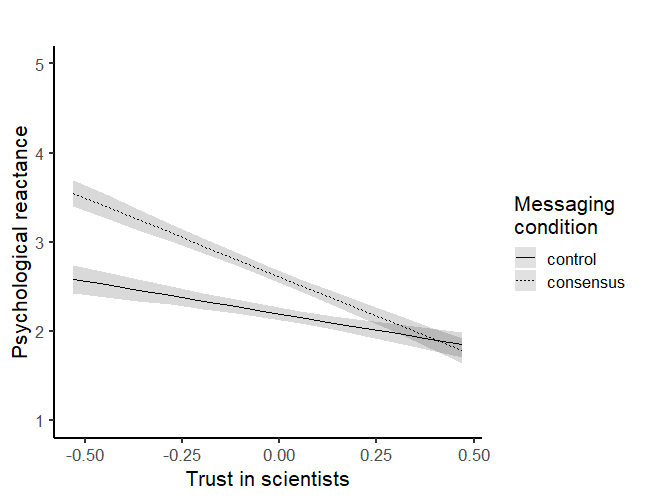


S7 Fig. The effects of trust in scientists and consensus messaging on psychological reactance in Study 3.


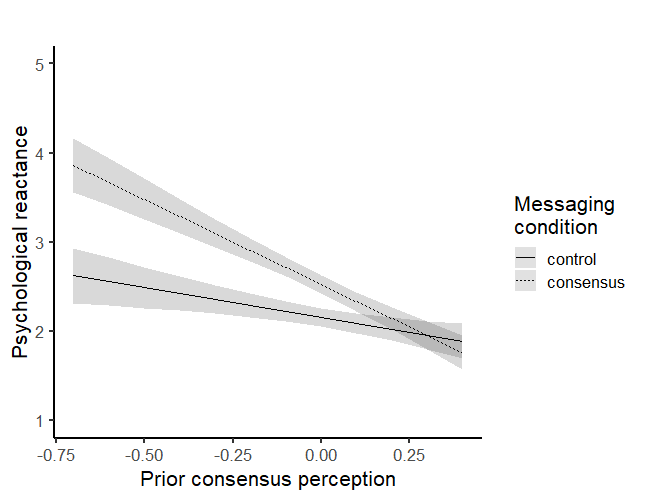


S8 Fig. The effects of trust in scientists and consensus messaging on psychological reactance in Study 3.

S31b Table. *The effects of consensus messaging and* ***moderators*** *on* ***psychological reactance*** *in Study 3 (subjects with low consensus perception and speeders are included; we do not control for demographic variables).*

|  | **Manipulations only** | | | **Ideology added** | | | **Trust added** | | | **Priors added** | | |
| --- | --- | --- | --- | --- | --- | --- | --- | --- | --- | --- | --- | --- |
|  | *b* | *SE* | *p* | *b* | *SE* | *p* | *b* | *SE* | *p* | *b* | *SE* | *p* |
| Intercept | 2.17 | 0.05 | **<0.001** | 2.18 | 0.05 | **<0.001** | 2.18 | 0.05 | **<0.001** | 2.20 | 0.05 | **<0.001** |
| Message [Consensus] | 0.39 | 0.06 | **<0.001** | 0.39 | 0.05 | **<0.001** | 0.39 | 0.05 | **<0.001** | 0.33 | 0.07 | **<0.001** |
| Design [Post-only] | 0.11 | 0.06 | 0.062 | 0.08 | 0.05 | 0.143 | 0.07 | 0.05 | 0.151 |  |  |  |
| Ideology |  |  |  | 0.92 | 0.21 | **<0.001** |  |  |  |  |  |  |
| Ideology x Message |  |  |  | 0.39 | 0.24 | 0.105 |  |  |  |  |  |  |
| Ideology x Design |  |  |  | 0.47 | 0.24 | 0.055 |  |  |  |  |  |  |
| Trust |  |  |  |  |  |  | -0.91 | 0.13 | **<0.001** |  |  |  |
| Trust x Message |  |  |  |  |  |  | -0.85 | 0.19 | **<0.001** |  |  |  |
| Prior perceived consensus |  |  |  |  |  |  |  |  |  | -1.00 | 0.20 | **<0.001** |
| Prior x Message |  |  |  |  |  |  |  |  |  | -0.82 | 0.29 | **0.004** |
| Observations | 1073 | | | 1073 | | | 1073 | | | 522 | | |
| R^2^ / R^2^ adjusted | 0.046 / 0.044 | | | 0.152 / 0.148 | | | 0.213 / 0.210 | | | 0.196 / 0.191 | | |

### **S3.4.2. The effects of psychological reactance and consensus messaging on the main DVs**

S32a Table. *The effects of* ***psychological reactance*** *and consensus messaging on the change scores in* ***key DVs*** *in Study 3 in the “pre-post” design condition.*

|  | **Perceived consensus** | | | **Worry** | | | **Belief** | | | **Policy support** | | | **Vax. int.** | | | **Boost. int.** | | |
| --- | --- | --- | --- | --- | --- | --- | --- | --- | --- | --- | --- | --- | --- | --- | --- | --- | --- | --- |
|  | *b* | *SE* | *p* | *b* | *SE* | *p* | *b* | *SE* | *p* | *b* | *SE* | *p* | *b* | *SE* | *p* | *b* | *SE* | *p* |
| Intercept | 3.28 | 2.93 | 0.264 | -0.07 | 0.07 | 0.341 | -0.04 | 0.08 | 0.634 | -0.05 | 0.04 | 0.241 | -0.02 | 0.07 | 0.796 | 0.00 | 0.11 | 0.991 |
| Age | -6.65 | 4.60 | 0.149 | 0.16 | 0.12 | 0.177 | 0.05 | 0.13 | 0.709 | 0.10 | 0.07 | 0.155 | -0.07 | 0.12 | 0.572 | 0.16 | 0.16 | 0.324 |
| Gender | 1.72 | 1.79 | 0.335 | -0.06 | 0.04 | 0.181 | 0.06 | 0.05 | 0.207 | -0.02 | 0.03 | 0.414 | -0.00 | 0.04 | 0.957 | 0.02 | 0.06 | 0.733 |
| Education | -1.60 | 3.09 | 0.605 | 0.05 | 0.08 | 0.519 | 0.00 | 0.09 | 0.960 | -0.00 | 0.05 | 0.981 | 0.02 | 0.08 | 0.765 | -0.04 | 0.10 | 0.684 |
| Message [Consensus] | 11.08 | 1.79 | **<0.001** | 0.02 | 0.04 | 0.686 | -0.00 | 0.05 | 0.945 | 0.01 | 0.03 | 0.829 | 0.04 | 0.05 | 0.355 | -0.12 | 0.06 | **0.045** |
| Psychological reactance | -1.53 | 6.18 | 0.804 | -0.09 | 0.16 | 0.574 | -0.03 | 0.18 | 0.860 | -0.06 | 0.09 | 0.557 | -0.03 | 0.15 | 0.834 | 0.68 | 0.21 | **0.002** |
| Message x Psych. react. | 7.13 | 7.85 | 0.364 | 0.11 | 0.20 | 0.575 | -0.00 | 0.23 | 0.986 | -0.21 | 0.12 | 0.075 | 0.06 | 0.20 | 0.764 | -0.96 | 0.27 | **<0.001** |
| Observations | 522 | | | 522 | | | 522 | | | 522 | | | 167 | | | 355 | | |
| R^2^ / R^2^ adjusted | 0.081 / 0.070 | | | 0.008 / -0.004 | | | 0.004 / -0.008 | | | 0.032 / 0.020 | | | 0.012 / -0.025 | | | 0.044 / 0.028 | | |

We found effects of psychological reactance and change scores on the intention to receive a booster shot of vaccine; the effects on other DVs were non-significant. The effects of psychological reactance on intention to receive a booster shot interacted with the messaging condition: the effect of psychological reactance was positive in the control message condition (b = 0.68, SE = 0.21, CI[0.26, 1.09]) but non-significant in the control condition (b = -0.29, SE = 0.18, CI[-0.64, 0.06]); the difference between the two slopes was statistically significant (b = 0.96, SE = 0.27, t(348) = 3.541, p = 0.001), see S9 Fig.


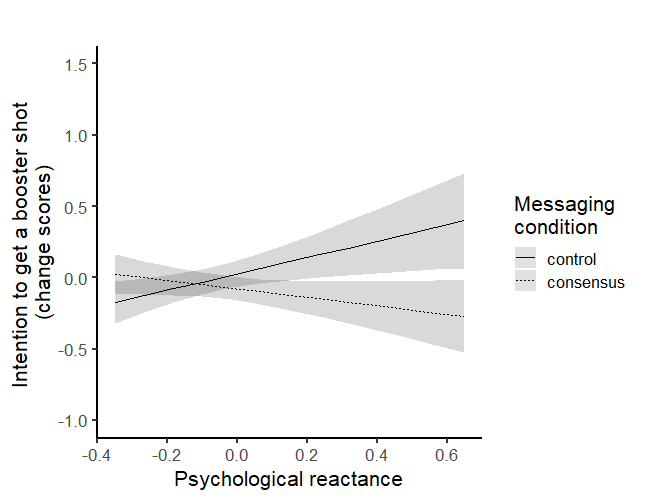


S9 Fig. The effects of psychological reactance and consensus messaging on the intention to receive a COVID-19 booster shot.

S32b Table. *The effects of* ***psychological reactance*** *and consensus messaging on the change scores in* ***key DVs*** *in Study 3 in the “pre-post” design condition (subjects with low consensus perception and speeders are included; we do not control for demographic variables).*

|  | **Perceived consensus** | | | **Worry** | | | **Belief** | | | **Policy support** | | | **Vax. int.** | | | **Boost. int.** | | |
| --- | --- | --- | --- | --- | --- | --- | --- | --- | --- | --- | --- | --- | --- | --- | --- | --- | --- | --- |
|  | *b* | *SE* | *p* | *b* | *SE* | *p* | *b* | *SE* | *p* | *b* | *SE* | *p* | *b* | *SE* | *p* | *b* | *SE* | *p* |
| Intercept | 1.03 | 1.29 | 0.425 | -0.02 | 0.03 | 0.521 | 0.01 | 0.04 | 0.715 | -0.03 | 0.02 | 0.117 | -0.02 | 0.03 | 0.400 | 0.05 | 0.04 | 0.307 |
| Message [Consensus] | 11.06 | 1.78 | **<0.001** | 0.02 | 0.04 | 0.673 | -0.00 | 0.05 | 0.955 | 0.01 | 0.03 | 0.844 | 0.05 | 0.05 | 0.316 | -0.13 | 0.06 | **0.040** |
| Psychological reactance | -1.28 | 6.12 | 0.834 | -0.09 | 0.15 | 0.537 | -0.04 | 0.18 | 0.827 | -0.05 | 0.09 | 0.575 | -0.05 | 0.14 | 0.721 | 0.67 | 0.21 | **0.001** |
| Message x Psychological reactance | 7.72 | 7.84 | 0.325 | 0.10 | 0.20 | 0.625 | -0.01 | 0.22 | 0.977 | -0.22 | 0.12 | 0.063 | 0.07 | 0.20 | 0.737 | -0.97 | 0.27 | **<0.001** |
| Observations | 522 | | | 522 | | | 522 | | | 522 | | | 167 | | | 355 | | |
| R^2^ / R^2^ adjusted | 0.076 / 0.071 | | | 0.001 / -0.005 | | | 0.000 / -0.005 | | | 0.026 / 0.020 | | | 0.009 / -0.009 | | | 0.040 / 0.032 | | |
