# Supplementary material for: Strong correlational but no causal evidence on the link between the perception of scientific consensus and support for vaccination
Source: PLoS One. 2024 Jan 3;19(1):e0296066. doi: 10.1371/journal.pone.0296066 (PMC10763927; doi:10.1371/journal.pone.0296066)
Supplement: Attachment — Submitted filename: response to reviewers.docx [file pone.0296066.s002.docx]

PONE-D-23-14532

Strong correlational but no causal evidence on the link between the perception of scientific consensus and support for vaccination

PLOS ONE

## **Editor’s comments**

Dear Dr. Czarnek,
Thank you for submitting your manuscript to PLOS ONE. After careful consideration, we feel that it has merit but does not fully meet PLOS ONE’s publication criteria as it currently stands. Therefore, we invite you to submit a revised version of the manuscript that addresses the points raised during the review process.

Thank you for your feedback and opportunity to revise our work.

E1. Kindly reanalyze and present all missing or deleted data to enable full appreciation by readers and future replication by other researchers, with particular reference to the following observations:

"Participants who indicated zero consensus". (a) Kindly give reasons for non-inclusion; (b). Rerun the analyses with these zero consensus participants included, and submit a comparative figure or table with those included; Or report these new results in the SI with a footnote referencing it in this section.

"Participants whose rating of the manipulation credibility is low". Kindly include these individuals’ responses into the analyses and report the result in the main text.

Removed "speeders (those below the 5 min)". Please add these participants’ results back in to *all* analyses, as well and update the result accordingly.

We repeated the analysis as recommended by yourself and Reviewers 2 and 3. In short, new analyzes did not change the results nor our conclusions. We specify what has been done for each study below.

Study 1

We repeated analysis with participants who indicated zero consensus. We also do not control for demographic variables in this analysis, as recommended in comment #E3 and by Reviewer 3. These results did not differ from the main analyzes in any meaningful way, i.e., the conclusions were not altered after re-analyzing the data.

We added a footnote mentioning these additional analysis (footnote 1, p. 7): “Re-analysis of the data with subjects reporting zero consensus and without controlling for demographic variables did not alter the results and conclusions. Details can be found in S1b, S3b, and S4b Tables.”

Study 2

We repeated analysis with zero consensus participants, those who perceived consensus manipulation as not credible, and speeders. We also do not control for demographic variables in this analysis.

There were no meaningful differences between the original and updated analysis. We have only recorded that the significant effect of condition on beliefs at wave 1 become non-significant in the updated analyses and that the effect of trust in scientists on policy support at wave 2 became also non-significant. We think these changes are very small and not worth reporting in the main text. This is because the paper is already long and adding these details will be only distracting.

We added a footnote mentioning these additional analysis (footnote 3, p. 11): “Re-analysis of the data with subjects reporting zero consensus, low credibility, speeders, and without controlling for demographic variables did not alter the results and conclusions. Details can be found in S5b, S6b, S7b, and S8b Tables).”

Study 3

We repeated analysis with zero consensus participants and speeders (those who perceived consensus manipulation as not credible were already included in the main analysis). Similarly to Studies 1 and 2, we also do not control for demographic variables in this analysis. We found only two small deviations from the original analyzes. These changes do not affect our conclusions in any way.

First, the interaction between consensus messaging and trust on policy support in the “post-only” design condition is now significant (b = -0.71, SE = 0.31, p = .023), whereas in the original analysis, this effect was non-significant at p = .074; see Supplementary Tables S21b and S21a, respectively. Nevertheless, pair-wise comparisons showed only a marginally significant trend for people low on trust in scientists: people in the consensus messaging condition (M = 2.06, SE = 0.08) had slightly higher support for the policies compared to the control condition control condition (M = 1.83, SE = 0.09); this difference was nevertheless only marginally significant (b = 0.23, SE = 0.12, t(547) = -1.94, p = 0.053). Given this difference is very small and only marginally significant, observed for only one DV, and in only one of the designs, it does not change our conclusions on the null effects of consensus messaging.

Second, the effects of the design condition on the psychological reactance became only marginally significant (b = 0.11, SE = 0.06, p = .062), whereas in the original analyses they were statistically significant; see Supplementary Tables S32b and S32a. Still, the originally reported effects of the design were rather small and against the sensitization hypothesis (higher in the post-only condition). Hence, the new findings do not change our conclusions that the design of the study was most likely not responsible for the null findings of the consensus manipulations.

We added a footnote mentioning the new analyzes (footnote 4, p. 17): “Re-analysis of the data with subjects reporting zero consensus, speeders, and without controlling for demographic variables did not alter the results and conclusions. Details can be found in S16b, S17b, S18b, S19b, S20b, S21b, S22b, S23b, S24b, S25b, S31b, and S32b Tables.”

Additionally, our results were not altered for SEM models. However, we decided not to report new SEM analyzes due to space and readability concern. Specifically, the Supplementary materials document is nearly 70-pages long and includes more than 60 tables). Given that the changes in the reported effects were none or minimal and our conclusions were not altered whatsoever, we do not think this reporting is necessary and will only further expand the already lengthy Supplementary materials.

Finally, we would like to highlight that removing participants who reported 0% consensus perception reported prior to manipulation was in line with our pre-registration for Studies 2 and 3. We added a note on p. (11), to briefly explain this decision: “In line with pre-registration, we removed (…) 11 subjects whose initial perception of scientific consensus was as low as 0% (as it suggests expressive responding).”

E2. Add a footnote indicating topics or 'unrelated items that were asked on the front end of studies 1 and 2', to give readers better clarity - and then if they wish they can go look up the specific items

We have added the requested information to footnotes. Specifically, we added:

- Footnote 2 (p. 8): “In Studies 1 and 2 these additional measures included perception of vaccines in general (not COVID-19 vaccine specifically), perceived social consensus regarding COVID-19 vaccines, COVID-19 threat, trust in institutions, political engagement, party preferences, need for cognitive closure (NFC). Further details are available in the pre-registrations.”
- Footnote 5 (p. 19): “In Study 3 the additional measures included perceived invulnerability to COVID-19 disease, threshold of scientific consensus necessary to trust the COVID-19 vaccine, perceived social consensus regarding COVID-19 vaccines, political engagement, party preferences, economic beliefs, NFC, and perceived threat related to war in Ukraine. Further details are available in the pre-registrations.”

E3.  Kindly report the results for the 4 out of 6 items that were not significant in the main section, and not the Supplementary material (SI).

We have added information about the requested results in Study 2. Additionally, we clarified the number of tests we have run. In the original manuscript we mention that the effects were non-significant for 4 out of 6 variables on interest. However, because we had two different consensus messaging conditions, in fact, we run 12 statistical tests, which we corrected now. The results description now states (p.15):

“These effects turned out to be non-significant for ten out of the 12 effects analyzed. Specifically, the effect of consensus messaging vs. control condition was non-significant for belief (b = 0.01, SE = 0.07, p = 0.896 at wave 2), worry (b = 0.05, SE = 0.06, p = 0.408 at wave 1; b = 0.05, SE = 0.08, p = 0.551 at wave 2) and policy support (b = -0.01, SE = 0.04, p = 0.848 at wave 1; b = -0.05, SE = 0.06, p = 0.443 at wave 2, Similarly, the effect of consensus and pluralism messaging vs. control condition was non-significant for belief (b = 0.03, SE = 0.05, p = 0.525 at wave 1; b = 0.12, SE = 0.07, p = 0.086 at wave 2), worry (b = 0.02, SE = 0.05, p = 0.703 at wave 1), and policy support (b = 0.06, SE = 0.04, p = 0.100 at wave 1; b = -0.03, SE = 0.06, p = 0.601 at wave 2).”

E4. Kindly include plots or statistical results for the final, key, and best run analysis in study 3.

We have added information about the requested results in Study 3 (p.21):

“Specifically, in the “pre-post” design condition the effects of consensus messaging on change scores in belief (b = <-0.01, SE = 0.04, p = 0.928), worry (b = -0.01, SE = 0.05, p = 0.920), policy support (b = -0.01, SE = 0.03, p = 0.835), vaccination intention with a first dose (b = -0.05, SE = 0.04, p = 0.243), or a booster shot (b = --0.06, SE = 0.06, p = 0.357) were non-significant. Similarly, in the “post-only” design condition the effects of consensus messaging on belief (b = 0.07, SE = 0.10, p = 0.466), worry (b = -0.09, SE = 0.09, p = 0.347), policy support (b = -0.09, SE = 0.11, p = 0.431), vaccination intention with a first dose (b = -0.10, SE = 0.14, p = 0.485), or a booster shot (b = -0.14, SE = 0.13, p = 0.273) were also non-significant.”

E5. Kindly Run models with and without demographic controls for all results and enter either both results into the relevant results section, or consistently report one set and then consistently just describe the results with language for the second set. Please also have the full models presented in the SI and a footnote to direct the reader

We have re-analyzed the data without the statistical control of demographic variables, as mentioned in our response to comment #E1 above. We decided to present the original analyzes in the main text as they are in line with our pre-registrations but present the new analyzes in the Supplementary materials. Removing statistical control of the demographic variables did not alter conclusions.

E6.  Furthermore, if there was any attrition for any reason, kindly presented at the same location as the sample size and make up. "That is, all studies should have a number that indicates the original sample collected at the start, the final number of participants who completed the study, and if these numbers are not the same, some indication of where these individuals attrited - this last point can be discussed in the SI (referenced by a footnote in the relevant location), but the first two numbers should be placed directly in the sample collection text."

We did not record any attrition throughout the experimental studies, except for the subjects in Study 2 who did not partake in wave 2. This has already been reported in the original manuscript but now added information about the attrition per condition at wave 2 (p. 11):

“(…) 754 people took part in the study at wave 1 (control condition N = 237, consensus condition N = 265, consensus & pluralism condition N = 252) and 587 at wave 2 control condition N= 183, consensus condition N= 209, consensus & pluralism condition N= 195; which corresponds to 77%, 78%, and, and 77% of the original sample per condition, respectively).”

E7. Consider including a correlation table that shows the correlations between all the variables mentioned (scientific consensus, worry, belief, policy support, ideology, etc.).

We added a correlation table with the key variables in Study 1 (p.8).

E8. Please consider relabeling the question “Does short messaging change the level of perception of the scientific consensus on vaccine safety and efficacy?” Perhaps this should be labelled as a "manipulation check"?

Thank you for this comment. We have nevertheless opted to keep the current headers for the tests of the effects of consensus messaging on consensus perceptions. There are two reasons for this. Firstly, in the original paper and the analysis of the consensus messaging, van der Linden and colleagues use change in consensus perception as a key part of the model (e.g., Kerr & van der Linden, 2021; van der Linden, Clarke, et al., 2015; van der Linden, Leiserowitz, et al., 2015). The model treats perception of consensus as a “gateway cognition”, thus, suggesting it is not a manipulation check but a key variable of interest. Secondly, the current title describes the analysis we have conducted. Hence, we think, it is easier for the readers to follow our analytical steps.

E9. Please address all other comments and observations raised by the peer-reviewers.

We have responded to comments below.

## **Reviewer #1:**

R1.1. This is an excellent manuscript describing several sound studies. My only issue with the study is that it treats trust in scientists as exogenous to COVID-19 attitudes and mitigation behaviors. I would suspect that people became less trusting of scientists as COVID-19 became politicized. However, the authors are consistent with the broader literature in treating it as exogenous. I wouldn't penalize these authors for something although maybe the authors can find work showing the dynamics or changes in trust toward scientists as a result of COVID-19.

Thank you for this comment. We added that comment to the General discussion section (p.24):

“Finally, while we treat trust in scientists as a moderator of the relationship between perceived consensus and vaccine attitudes, it is also likely that trust in scientists changes an issue becomes increasingly politicized [32,66] or consensus messaging itself might change perception of scientists [67].”

32. Gauchat G. Politicization of Science in the Public Sphere: A Study of Public Trust in the United States, 1974 to 2010. Am Sociol Rev. 2012;77: 167–187. doi:10.1177/0003122412438225

66. Oreskes N, Conway EM. From Anti-Government to Anti-Science: Why Conservatives Have Turned Against Science. Daedalus. 2022;151: 98–123. doi:10.1162/daed_a_01946

67. Orchinik R, Dubey R, Gershman S, Powell D, Bhui R. Learning About Scientists from Climate Consensus Messaging. Proceedings of the Annual Meeting of the Cognitive Science Society. 2023. Available: https://escholarship.org/uc/item/0x36617d

R1.2. In regards to the broader project, more integration of norms would be helpful. The authors focus on consensus at the elite level, but counter views existed creating in-group norms among other elites or peers. Future work should consider this dynamic.

Thank you for this comment. The Gateway Beliefs Model, a theory that we test in this research, focuses specifically on perception of expert or elite consensus and does not account for the impact of ingroup norms. We also acknowledge that focus on expert consensus have an important practical implications, given the polarized context of vaccination. However, we agree that the perceptions of social norms indeed shapes attitudes and behavior. Hence, we added a note in the General discussion section (p.24):

“Additionally, it is worth noting that while the abovementioned research focuses on the effects of expert consensus, whether medical practitioners or scientists, it is likely that perception of social norms might influence vaccine attitudes and intentions. In this context especially relevant are norms within closest social circle and political in-groups [65].”

65. Rabb N, Bowers J, Glick D, Wilson KH, Yokum D. The influence of social norms varies with “others” groups: Evidence from COVID-19 vaccination intentions. Proc Natl Acad Sci. 2022;119: e2118770119. doi:10.1073/pnas.2118770119

## **Reviewer #2:**

The authors of the paper run a correlational study and two survey experiments evaluating the relationship between scientific consensus about vaccines, vaccination attitudes, and intentions. The research is thorough and addresses a question of high practical importance. I think this work would fit well in PLOS ONE.

There are a few changes that I would like to suggest:

R2.1. Because Study 1 is a correlational study, it would be helpful to include a correlation table that shows the correlations between all the variables mentioned (scientific consensus, worry, belief, policy support, ideology, etc.). It makes it difficult to interpret the results when there is no correlation table.

We added a correlation table with the key variables in Study 1 (p.8).

R2.2. In Table 1, the second question is: “Does short messaging change the level of perception of the scientific consensus on vaccine safety and efficacy?” However, this seems more of a manipulation check than a research question. I would re-label this as a manipulation check.

Thank you for this comment. We have decided to keep the current headers and questions in the table. As mentioned in response to comment #E2, there are two reasons for this decision. Firstly, in the original paper and the analysis of the consensus messaging, van der Linden and colleagues use change in consensus perception as a key part of the model (e.g., Kerr & van der Linden, 2021; van der Linden, Clarke, et al., 2015; van der Linden, Leiserowitz, et al., 2015). The model treats perception of consensus as a “gateway cognition”, thus, suggesting it is not a manipulation check but a key variable of interest. Secondly, the current title describes the analysis we have conducted. Hence, we think, it is easier for the readers to follow our analytical steps.

R2.3. In Study 2, 134 participants were dropped for low ratings of manipulation credibility. Is this post-treatment? If so, then differential attrition could explain the results. Please indicate if this is pre or post treatment, and if post-treatment, it would be helpful to run a robustness check including these respondents.

In line with this suggestion, we re-analyzed the data with inclusion of subjects who perceived consensus messaging manipulation as low on credibility. In short, it did not change our conclusions. The details of these analyzes are provided in response to comment #E1.

R2.4. I do not see the pre-registration files in the OSF. Please include links to the pre-registrations in the manuscript.

Thank you for noticing the missing files. We now made the OSF project publicly available and replaced the link to the anonymized project with this one: <https://osf.io/dnjbu/>. The pre-registrations are available under “pre-registration” tab. Additionally, we added direct pre-registration links to each study Method section.

Study 1 <https://osf.io/35pn6>

Study 2 <https://osf.io/myegr>

Study 3 <https://osf.io/hwm6k>

R2.5. One reason for the null effects may be that for a salient issue like COVID, a one-sentence message is simply not strong enough to change behaviors. It would be helpful to add this as a limitation.

Thank you for this comment. A key prediction of the Gateway Beliefs Model (GBM), that we test in this research, is that changing perception of expert consensus changes in associated attitudes and behavioral tendencies. However, while we successfully lifted perception of consensus, it was unrelated to changes in associated attitudes or behavioral intentions.

While we agree in principle with this comment that maybe one short messaging is not strong enough to change prior beliefs, the GBM also predicts that the impact on beliefs should be largest in magnitude but smaller on behavior. This is also was not supported by the current study as the effects of different versions of consensus messaging (consensus or consensus & plurality in Study 2; consensus in pre-post vs. post-only design in Study 3) all yielded null effects. This

We nevertheless added a note (also inspired by comments #1.1 and #1.2 from Reviewer 1) highlighting that that perception of expert or social norms, and their dynamics, could have an impact on the null findings.

“Furthermore, it is worth noting that while the abovementioned research focuses on the effects of expert consensus, whether medical practitioners or scientists, it is likely that perception of social norms might influence vaccine attitudes and intentions. In this context especially relevant are norms within closest social circle and political in-groups [65]. Finally, while we treat trust in scientists as a moderator of the relationship between perceived consensus and vaccine attitudes, it is also likely that trust in scientists changes an issue becomes increasingly politicized [32,66] or consensus messaging itself might change perception of scientists [67]. The question of what is the dynamics of consensus messaging effectiveness is could be an intriguing avenue for future research.”

32. Gauchat G. Politicization of Science in the Public Sphere: A Study of Public Trust in the United States, 1974 to 2010. Am Sociol Rev. 2012;77: 167–187. doi:10.1177/0003122412438225

65. Rabb N, Bowers J, Glick D, Wilson KH, Yokum D. The influence of social norms varies with “others” groups: Evidence from COVID-19 vaccination intentions. Proc Natl Acad Sci. 2022;119: e2118770119. doi:10.1073/pnas.2118770119

66. Oreskes N, Conway EM. From Anti-Government to Anti-Science: Why Conservatives Have Turned Against Science. Daedalus. 2022;151: 98–123. doi:10.1162/daed_a_01946

67. Orchinik R, Dubey R, Gershman S, Powell D, Bhui R. Learning About Scientists from Climate Consensus Messaging. Proceedings of the Annual Meeting of the Cognitive Science Society. 2023. Available: https://escholarship.org/uc/item/0x36617d

## **Reviewer #3:**

Overall this is a thoughtful test of the role of individual perceptions of the scientific consensus on reported willingness to get vaccinated - in the contexts of Poland. The authors found that, though there is clear evidence for a correlational link between attitudes of scientific consensus and willingness to get vaccinated, there appears to be no causal link, as evidenced by the authors ability to move the former but not the later with a series of experimental studies using consensus messaging. Overall I am positive about this manuscript and will suggest publication once the following changes have been made.

R3.1. On line 127 the authors indicate that they removed participants who indicated zero consensus. A reason was not given, but I assume it's because the authors believed these individuals were not paying attention or were being insincere in their answer? It could also be that they were engaging in some form of expressive responding. What do the results look like with these individuals added? It seems like this would be the cleanest way of running the analysis - not top down filtering. Though removing these individuals may work against the authors’ position, it still seems like not the cleanest choice.
The authors should:
1- Indicate their reasoning for this decision.
2- Rerun the analyses with these zero consensus participants included
3- And then report these new results in the SI with a footnote referencing it in this section.

Similarly, on line 269 you remove participants whose rating of the manipulation credibility is low, but that’s a violation of randomization and messes with internal validity. That is, these individuals easily could be very low on trust in scientists, trust in the vaccine or both - which could be why they think the message is low. So removing them both violates random assignment but also (and again) works against the authors’ conclusion.
The authors also seem to note this in the intro to study 3, but strangely don't seem to just change the analysis even though (I assume) they have the data?
The authors should:
1- include these individuals’ responses into the analyses and report the result in the main text

Also, removing speeders (those below the 5 min) is a failure of random assignment for the same above reason.
1- Please add these participants’ results back in to *all* analyses, as well and update the results.

Thank you for these comments. In line with the Reviewer’s suggestions, we re-analyzed the data with inclusions of speeders (Studies 1-3), zero consensus participants (Studies 2-3), and subjects with low credibility perception (Study 2). In response to comment #3.6 below, we also did not control for the demographic variables. In short, it did not change our conclusions. The details of these analyzes are provided in response to comment #E1.

R3.2. The authors indicate several times that other, unrelated items were asked on the front end of studies 1 and 2 and then point to the pre-registration. I think it's fair to say that almost no one will look at this and the authors should instead:
1- add in a footnote the topic of the items that were asked to give the reader some easy understanding - and then if they wish they can go look up the specific items

We have added requested information to footnotes. Specifically:

- Footnote 2 (p. 8): “In Studies 1 and 2 these additional measures included perception of vaccines in general (not COVID-19 vaccine specifically), perceived social consensus regarding COVID-19 vaccines, COVID-19 threat, trust in institutions, political engagement, party preferences, need for cognitive closure (NFC). Further details are available in the pre-registrations.”
- Footnote 5 (p. 19): “In Study 3 the additional measures included perceived invulnerability to COVID-19 disease, threshold of scientific consensus necessary to trust the COVID-19 vaccine, perceived social consensus regarding COVID-19 vaccines, political engagement, party preferences, economic beliefs, NFC, and perceived threat related to war in Ukraine. Further details are available in the pre-registrations.”

R3.3. On line 363 the authors note that 4 out of 6 of the results were not sig, but they do not report them and only report on the two that were significant.
The authors should:
1- Report the results for the 4 that were not sig in the main section, not the SI

We have added information about the requested results in Study 2. Additionally, we clarified the number of tests we have run. In the original manuscript we mention that the effects were non-significant for 4 out of 6 variables on interest. However, because we had two different consensus messaging conditions, in fact, we run 12 statistical tests, which we corrected now. The results description now states (p.15):

“These effects turned out to be non-significant for ten out of the 12 effects analyzed. Specifically, the effect of consensus messaging vs. control condition was non-significant for belief (b = 0.01, SE = 0.07, p = 0.896 at wave 2), worry (b = 0.05, SE = 0.06, p = 0.408 at wave 1; b = 0.05, SE = 0.08, p = 0.551 at wave 2) and policy support (b = -0.01, SE = 0.04, p = 0.848 at wave 1; b = -0.05, SE = 0.06, p = 0.443 at wave 2, Similarly, the effect of consensus and pluralism messaging vs. control condition was non-significant for belief (b = 0.03, SE = 0.05, p = 0.525 at wave 1; b = 0.12, SE = 0.07, p = 0.086 at wave 2), worry (b = 0.02, SE = 0.05, p = 0.703 at wave 1), and policy support (b = 0.06, SE = 0.04, p = 0.100 at wave 1; b = -0.03, SE = 0.06, p = 0.601 at wave 2).”

R3.4. I do think that the same in S2 was a bit small - for a test between two means and using the original same size makes sense, but still the results do appear like they would be sig with a larger sample.

We used power analysis to determine minimum sample size and the sample we used was of similar size to a study that reported significant effects of consensus manipulation (i.e., Kerr & van der Linden, 2021). Even if the sample fall short in some sense, the additional evidence for the null consensus messaging effects was presented in Study 3.

R3.5. It's somewhat strange that the authors did not include plots or statistical results for the final, key, and best run analysis in study 3.
The authors need to:
1- add in these results to make it easier for the reader to understand what they have done
2- they should also include a plot as with all the other key results

We have added information about the requested results in Study 3 (p.21):

“Specifically, in the “pre-post” design condition the effects of consensus messaging on change scores in belief (b = <-0.01, SE = 0.04, p = 0.928), worry (b = -0.01, SE = 0.05, p = 0.920), policy support (b = -0.01, SE = 0.03, p = 0.835), vaccination intention with a first dose (b = -0.05, SE = 0.04, p = 0.243), or a booster shot (b = --0.06, SE = 0.06, p = 0.357) were non-significant. Similarly, in the “post-only” design condition the effects of consensus messaging on belief (b = 0.07, SE = 0.10, p = 0.466), worry (b = -0.09, SE = 0.09, p = 0.347), policy support (b = -0.09, SE = 0.11, p = 0.431), vaccination intention with a first dose (b = -0.10, SE = 0.14, p = 0.485), or a booster shot (b = -0.14, SE = 0.13, p = 0.273) were also non-significant.”

R3.6. Overall, these models are compelling (my critiques above withstanding); however, there are analyses in the SI that seem to use demographic controls in the model - I would request that the authors:
1- Run models with and without demographic controls for all results
2- Enter either both results into the relevant results section, or consistently report one set and then consistently just describe the results with language for the second set
3- have the full models presented in the SI and a footnote to direct the reader

As mentioned in response to comment #R3.1, we repeat the analyzes as requested by the Reviewer and our conclusions did not change. In the main paper we report the findings from the analysis that includes demographic controls as such analyzes were pre-registered.

R3.7. Lastly, if there was any attrition for any reason, I will request these numbers be presented at the same location as the sample size and make up. That is, all studies should have a number that indicates the original sample collected at the start, the final number of participants who completed the study, and if these numbers are not the same, some indication of where these individuals attrited - this last point can be discussed in the SI (referenced by a footnote in the relevant location), but the first two numbers should be placed directly in the sample collection text.

We did not record any attrition throughout the experimental studies, except for the subjects in Study 2 who did not partake in wave 2. This has already been reported in the original manuscript but now added information about the attrition per condition at wave 2 (p.11):

“(…) 754 people took part in the study at wave 1 (control condition N = 237, consensus condition N = 265, consensus & pluralism condition N = 252) and 587 at wave 2 control condition N= 183, consensus condition N= 209, consensus & pluralism condition N= 195; which corresponds to 77%, 78%, and, and 77% of the original sample per condition, respectively).”
